# VAEBAC: a representation-learning framework for proteome-scale prediction of amyloidogenic proteins and functional stratification of nucleic-acid-binding proteins

**DOI:** 10.1093/bioinformatics/btag745

**Published:** 2026-10-07

**Authors:** Muhammad Uzair Ashraf, Owais Ahmad, Rizwan Hasan Khan

**Affiliations:** Interdisciplinary Biotechnology Unit, Faculty of Life Sciences, Aligarh Muslim University, Aligarh, Uttar Pradesh 202002, India; Interdisciplinary Biotechnology Unit, Faculty of Life Sciences, Aligarh Muslim University, Aligarh, Uttar Pradesh 202002, India; Interdisciplinary Biotechnology Unit, Faculty of Life Sciences, Aligarh Muslim University, Aligarh, Uttar Pradesh 202002, India

## Abstract

**Motivation:**

Nucleic-acid-binding proteins (NABPs) govern transcription, replication and genome organization, yet the distribution of aggregation susceptibility within regulatory proteomes remains unresolved. Here, we systematically examine amyloidogenic potential across the nucleic-acid-binding proteomes of four phylogenetically diverse organisms—*Escherichia coli*, *Bacillus subtilis*, *Saccharomyces cerevisiae*, and *Homo sapiens*—spanning prokaryotes and eukaryotes, to determine whether aggregation-prone sequence architectures are selectively constrained within transcriptional networks.

**Results:**

Using VAEBAC, a representation-learning framework that integrates large-scale amyloid-family sequences with experimentally validated annotations, we performed proteome-scale prediction of amyloidogenicity across nucleic-acid-binding proteins in each organism. Predicted amyloidogenic proteins were consistently and significantly enriched in Gene Ontology categories associated with regulation of DNA-templated transcription, RNA biosynthesis, and gene expression across all four organisms, a pattern validated using three independent annotation tools. Fisher’s exact test confirmed significant enrichment of amyloidogenic NABPs among transcription regulators in eukaryotic proteomes. Residue-level mapping demonstrated spatial separation between predicted aggregation-prone regions and DNA-binding interfaces, suggesting structural compatibility between regulatory function and conditional aggregation. Experimental validation confirmed fibrillar assembly of selected proteins. These findings support a conserved model in which aggregation susceptibility is functionally stratified across transcriptional regulatory tiers rather than uniformly suppressed within NABPs, a principle conserved across 3.5 billion years of evolution.

**Availability and implementation:**

VAEBAC is freely accessible at https://www.vaebac.com.

## Graphical Abstract

**Graphical Abstract:**
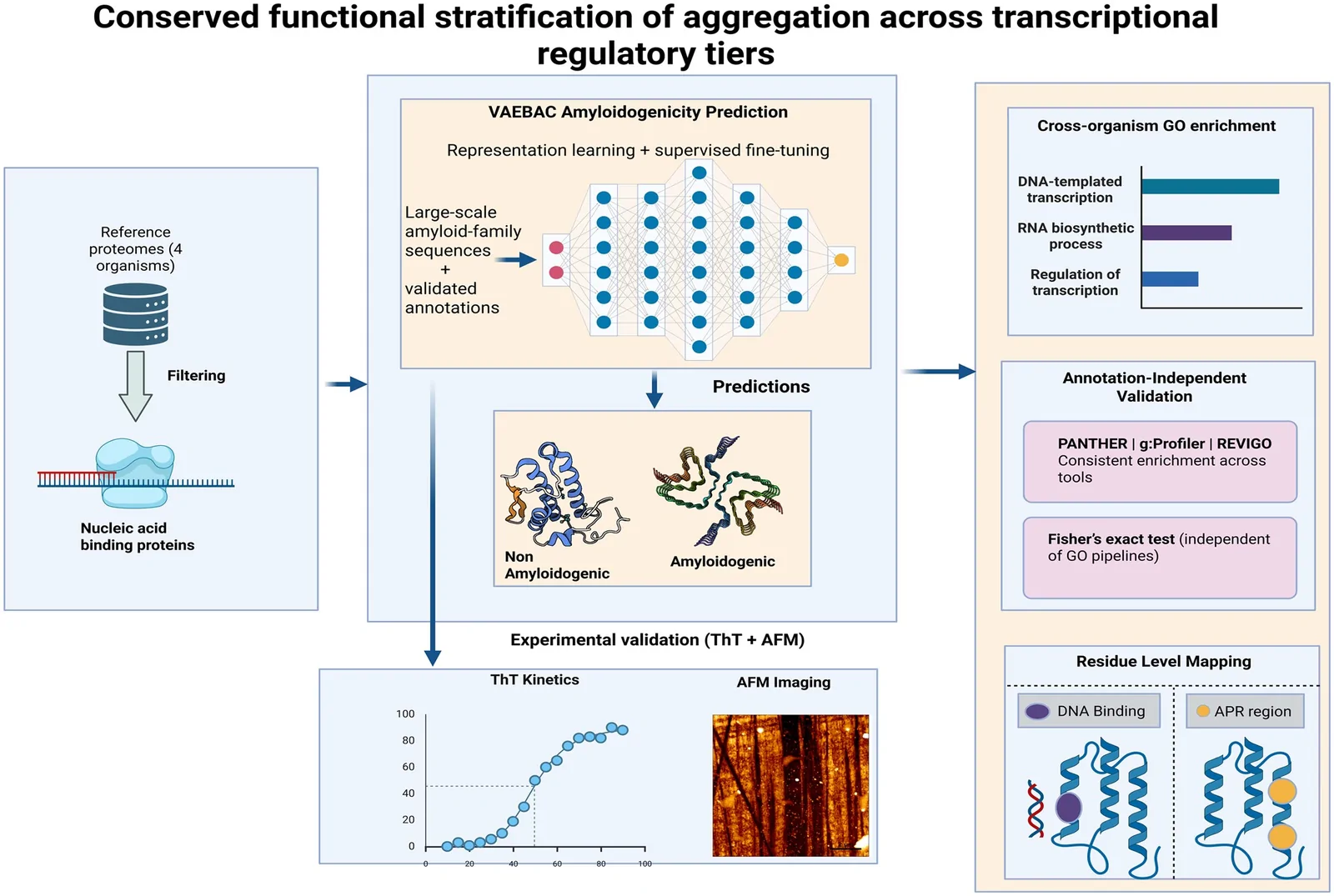

## 1 Introduction

Proteins that bind to DNA or RNA (nucleic-acid-binding proteins, NABPs) play critical roles in transcription, replication, repair, and other genetic processes (Hudson and Ortlund 2014, Hentze *et al.* 2018). These NABPs often have intrinsically disordered or basic regions that promote nucleic acid interactions but are also prone to misfolding that may eventually lead to amyloid aggregation (Wright and Dyson 2015). Amyloid aggregates are insoluble fibrillar assemblies having a typical cross-β structure. Although best known from eukaryotic neurodegenerative diseases, research has shown that amyloids can also occur as similar cross-β assemblies in bacteria (Krishnan and Lindquist 2005, Knowles *et al.* 2014, Otzen and Riek 2019). Crucially, many bacterial amyloids serve distinct functional roles (Van Gerven *et al.* 2018). Documented roles span control of chromosome replication timing, biofilm matrix architecture, surface attachment, and virulence-associated interactions with the host (Kosolapova *et al.* 2020, Akbey and Andreasen 2022, Bloch *et al.* 2025). Interestingly, a hallmark of bacterial functional amyloids is their interaction with nucleic acids. For example, the *E. coli* RNA chaperone Hfq has an intrinsically disordered C-terminal tail that assembles into an amyloid-like polymer to bridge and compact DNA (Vogel and Luisi 2011, Cech *et al.* 2016, Caillet *et al.* 2019). Similarly, during stress conditions in *E. coli*, Dps-DNA complexes accumulate in the nucleoid. The starvation-induced DNA-binding protein Dps forms large insoluble assemblies with chromosomal DNA (Martinez and Kolter 1997, Karas *et al.* 2015). These examples show that the aggregation of nucleoid-binding proteins can directly affect genome structure and gene regulation. Recent studies suggest that these aggregation events are not just a result of stress. Conditional aggregation of specific proteins can help change transcriptional programs or redirect cellular resources during stress adaptation (Saarikangas and Barral 2016, Fassler *et al.* 2021). In this context, the tendency to aggregate may be a trait that influences transcription accuracy and the structure of regulatory networks in changing environments.

A wealth of computational methods have been developed to predict protein aggregation or amyloid-forming segments from sequence. Early computational predictors of amyloidogenicity mainly focused on detecting short aggregation-prone sequence segments with high β-sheet propensity (Fernandez-Escamilla *et al.* 2004, Frousios *et al.* 2009). While these approaches perform well for short peptides, they tend to overpredict amyloidogenicity when applied to full-length proteins, as most proteins can have multiple local aggregation-prone motifs, thereby failing to capture long-range sequence context and global structural effects (Goldschmidt *et al.* 2010, Wilson *et al.* 2018, Louros *et al.* 2020). More importantly, most existing aggregation predictors have been trained predominantly on disease-associated or structural amyloids and do not explicitly account for the sequence architecture of transcriptional regulators or other nucleic-acid-binding proteins, which remain underrepresented in standard aggregation training datasets (Pawlicki *et al.* 2008, Redler *et al.* 2014, Navarro and Ventura 2022). This limitation has hindered investigation of how aggregation susceptibility is distributed across nucleic-acid-binding proteomes and whether aggregation-prone sequence architectures are selectively tolerated within specific transcriptional regulatory tiers. Accordingly, reliable proteome-scale assessment of amyloidogenicity within nucleic-acid-binding proteins is therefore required to determine whether aggregation susceptibility is functionally constrained within transcriptional regulatory networks.

With the rise of machine learning and deep learning algorithms, several efforts have been made to develop new and effective methods for classifying whole-protein sequences based on their likelihood of forming amyloids. For instance, the AmyloGram tool encodes protein sequences using n-grams and trains a Random Forest classifier. This method successfully classifies annotated amyloid proteins with high confidence (Burdukiewicz *et al.* 2017). Similarly, PredAmyl-MLP combined multiple sequence-derived features with a multilayer perceptron, reaching about 91.6% cross-validated accuracy and surpassing earlier methods (Li *et al.* 2020). The ENTAIL classifier used thousands of molecular descriptors along with a Naive Bayes kernel model to achieve ∼ 81.8% accuracy with MCC around 0.68 on a balanced amyloid/non-amyloid dataset (Auriemma Citarella *et al.* 2022). A recent meta-predictor, AMYPred-FRL (Charoenkwan *et al.* 2022), fused multiple machine learning algorithms over probabilistic sequence features, attaining ∼87.3% accuracy and MCC ∼ 0.71. These studies demonstrate that well-engineered machine-learning classifiers can improve upon consensus scoring approaches; however, their architectures are typically shallow due to the limited size of curated training datasets, which remains a primary challenge for employing deep learning in amyloidogenicity prediction. At present, experimentally validated amyloidogenic protein datasets, such as AmyPro (Varadi *et al.* 2018), remain limited in size, imposing substantial constraints on supervised model development and increasing the risk of overfitting in high-capacity architectures. Owing to this limited availability of experimentally validated sequences, we adopted a representation-learning strategy that leverages both large-scale unannotated data and high-confidence experimental annotations to enable robust classification of aggregation propensity within nucleic-acid-binding proteins. We first utilized AMYPdb (Pawlicki *et al.* 2008), a publicly available database comprising 9181 amyloid-family protein entries that, while not experimentally validated, collectively capture the broader sequence diversity characteristic of amyloidogenic proteins. This dataset was employed to train a variational autoencoder (VAE) in an unsupervised manner, enabling the model to learn a compact latent representation of amyloid-related sequence features without reliance on experimental labels. Following convergence, the encoder was frozen to preserve the learned sequence representations, and the decoder was replaced with a lightweight multilayer perceptron (MLP). The resulting model, termed VAEBAC (Variational Autoencoder–Based Amyloid Classifier), was subsequently fine-tuned using experimentally validated amyloidogenic proteins curated in the AmyPro database as positive labels.

Application of VAEBAC to the nucleic-acid-binding proteomes of four organisms spanning prokaryotes and eukaryotes (Table 1) revealed conserved evidence of functional stratification in aggregation propensity. Amyloidogenic NABPs were consistently enriched within transcriptional regulatory categories across all organisms examined, suggesting that aggregation susceptibility may be a selectively tolerated feature of transcriptional regulatory networks rather than a uniformly suppressed liability.

**Table 1:** Summary of VAEBAC-predicted amyloidogenic and non-amyloidogenic nucleic-acid-binding proteins across four organisms.^a^

Organism	Total NABPs	Amyloidogenic	Non-amyloidogenic	% Amyloidogenic
*E. coli*	772	6	766	0.8
*B. subtilis*	568	13	555	2.3
*S. cerevisiae*	1264	31	1233	2.5
*H. sapiens*	4005	189	3816	4.7

a All NABPs were retrieved using GO:0003676 (any evidence code, reviewed Swiss-Prot entries) and classified at a uniform threshold of 0.95.

## 2 Materials and methods

### 2.1 Datasets

The AMYPdb (Pawlicki *et al.* 2008) database was used for unsupervised pretraining of the VAE. The dataset comprises 9181 amyloid-family protein sequences curated based on sequence homology and functional annotation but not experimentally validated for amyloidogenicity. All AMYPdb sequences were used exclusively for representation learning and were not included in downstream supervised training or evaluation.

The benchmark dataset utilized for supervised model training and evaluation was derived from the Amy dataset initially compiled by Niu *et al.* (2018). This dataset has been widely utilized in the creation and evaluation of various amyloid prediction methodologies, including RFAmyloid (Niu *et al.* 2018), iAMY-SCM (Charoenkwan *et al.* 2021), AMYPred-FRL (Charoenkwan *et al.* 2022), and FA-Amy (Wang *et al.* 2026), facilitating a standardized performance comparison among different existing models. The Amy dataset was produced by combining empirically supported amyloidogenic protein sequences from the AmyPro database (Varadi *et al.* 2018) with non-amyloid entries from UniProt (UniProt Consortium 2023). CD-HIT (Fu *et al.* 2012) was then used to eliminate sequences with high mutual similarity and reduce redundancy, helping to eliminate bias from closely related sequences. Before training the model, any ambiguous residues were removed from the sequences. There were 9181 items in the AMYPdb dataset, but one sequence with missing values was dropped; therefore the final dataset included 9180 sequences. To preserve class balance, the dataset was split into training, validation, and test sets using a stratified sampling method in scikit-learn (Pedregosa *et al.* 2011). Cross-validation results on the training set are summarized in Table 2.

**Table 2:** Evaluation metrics on the training dataset under stratified five-fold and ten-fold cross-validation.^a^

CV Scheme	**Accuracy (mean** ± **SD)**	**MCC (mean** ±**SD)**	**AUC (mean** ± **SD)**
Five-Fold CV	0.875±0.021	0.713±0.054	0.944±0.019
Ten-Fold CV	0.878±0.019	0.721±0.043	0.943±0.030

a Values represent mean ± SD across folds.

To directly compare against a widely-used alternative sequence representation, we generated position-specific scoring matrices (PSSM) via PSI-BLAST (Altschul *et al.* 1997) (three iterations, E-value < 0.001) against the Swiss-Prot database (UniProt Consortium 2023) for all training and independent test sequences; 56 of 518 sequences (47 negative, 9 positive) yielded no detectable homologs at this threshold and were excluded from this comparison (Table 8, available as supplementary data at *Bioinformatics* online).

### 2.2 Sequence embedding and representation

Protein sequences were encoded using two complementary residue-level representations designed to capture both categorical identity and physicochemical characteristics. First, amino acid sequences were represented using one-hot encoding, preserving the discrete identity of each residue. In parallel, a physicochemical feature-based representation was constructed for each residue, comprising hydrophobicity (Kyte–Doolittle scale) (Kyte and Doolittle 1982), net charge (Uversky *et al.* 2000), solvent accessibility (Tien *et al.* 2013), and β-sheet propensity (Chou and Fasman 1974).

All physicochemical features were independently normalized to the range [0, 1] using Min–Max normalization. The one-hot and physicochemical representations were generated separately and used as distinct input feature sets in downstream modeling.

### 2.3 VAEBAC architecture

The proposed framework consists of a VAE-based encoder trained to learn latent representations of amyloid-related protein sequences, followed by a task-specific classifier for amyloidogenicity prediction. As illustrated schematically (Fig. 1), protein sequences were processed using two parallel representation streams corresponding to categorical and physicochemical encodings.

**Figure 1:**
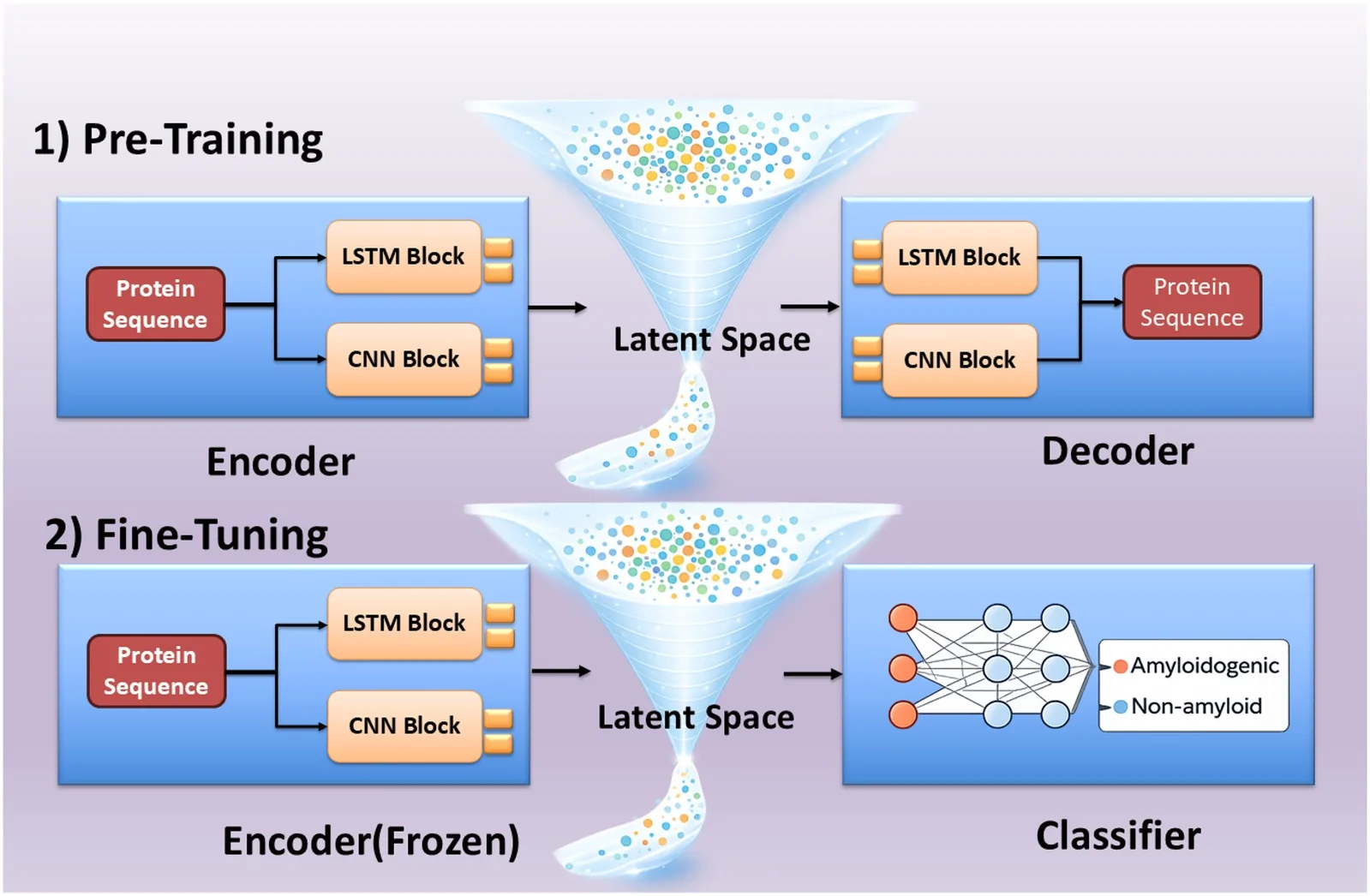
Architecture of the VAEBAC framework for amyloidogenicity prediction. Protein sequences are encoded using two parallel representations: one-hot encoding capturing residue identity and physicochemical feature vectors describing hydrophobicity, charge, solvent accessibility, and β-sheet propensity. These representations are processed by a VAE to learn a latent sequence representation during unsupervised pretraining on the AMYPdb dataset. For supervised prediction, the pretrained encoder is frozen and coupled to a multilayer perceptron classifier trained on experimentally validated amyloidogenic proteins from the AmyPro dataset.

The one-hot-encoded sequence representation was used to capture sequential dependencies along the protein chain, while the physicochemical feature representation was designed to capture local compositional and structural tendencies. These two representations were processed independently within the encoder and subsequently integrated to form a unified latent representation. To accommodate proteins of varying length within a fixed-dimensional representation, sequences were zero-padded to a single fixed maximum length of 10 746 residues (the longest sequence in the pretraining corpus) prior to encoding, an approach analogous to the zero-vector padding of full-length evolutionary profiles used by Chauhan and Ahmad (2020). Both input streams were processed by recurrent (LSTM-based) sub-networks operating on packed-sequence representations, so that padded positions are excluded from the recurrence itself; the resulting hidden states were projected through a shared 1×1 convolution, concatenated channel-wise, and reduced to a fixed-dimensional vector by adaptive average pooling.

We note explicitly that this pooling step is the point at which sequence length enters the latent representation. Because the average is taken across the full padded width of 10 746 positions rather than across the true sequence length, each pooled activation is approximately the sum of its per-residue contributions scaled by (true length/10 746). The pooled representation is therefore fixed-*dimensional*, but it is not invariant to sequence length: length is encoded in the latent space by construction. This is the mechanistic origin of the length-correlated signal we characterize in Results and quantify in Tables 12 and 13 and Figs 14–19, available as supplementary data at *Bioinformatics* online, and we draw attention to it here rather than leaving it to be inferred from the interpretability analyses.

During the unsupervised pretraining phase, the encoder was coupled to a decoder network to form a VAE, enabling learning of a compact latent space that captures general amyloid-family sequence characteristics. The decoder was trained to reconstruct the input sequence representation from the latent space.

For supervised amyloidogenicity prediction, the pretrained encoder was frozen and the decoder was replaced with a lightweight multilayer perceptron (MLP) classifier. The classifier was trained on experimentally validated amyloidogenic proteins to map the learned latent representations to binary output labels. Detailed architectural specifications for the encoder, decoder, and classifier networks are provided in Tables 1 and 2, available as supplementary data at *Bioinformatics* online.

VAEBAC produces a continuous amyloidogenicity score in [0, 1]; consequently, binary metrics such as accuracy, sensitivity, specificity, and MCC depend on the decision threshold selected, and different thresholds trace out different points along the same ROC curve. We therefore report two explicitly defined operating points on the independent benchmark (Table 3, Fig. 3), in addition to the threshold-independent AUC. The first is the Youden J-optimal threshold (τ=0.43919), obtained by maximizing sensitivity + specificity –1 on the independent benchmark itself; this yields a balanced operating point (sensitivity 0.848, specificity 0.844). The second is a benchmark-calibrated threshold (τ=0.9354), selected from the same independent benchmark to provide the operating point used for the subsequent external evaluation. The corresponding confusion matrices and threshold-dependent metrics are reported explicitly for these two operating points. Importantly, the benchmark-calibrated threshold of τ=0.9354 was fixed before evaluation on the Yang *et al.* dataset and was applied unchanged, with no threshold optimization performed on the external dataset. Thus, the Yang *et al.* evaluation represents an external application of a previously fixed operating threshold. AUROC, being threshold-independent, is also reported as the principal measure of discrimination. The Methods, Table 3 caption, and Fig. 3 legend explicitly identify the two operating points and the corresponding threshold-dependent metrics. For proteome-scale inference, a separate, more stringent operating threshold of 0.95 was fixed *a priori* (i.e. not derived from any evaluation dataset) to minimize false-positive predictions, which can accumulate substantially in large proteomes and bias downstream enrichment analyses. This higher threshold places the model in a high-specificity operating regime, ensuring that identified amyloidogenic candidates represent high-confidence predictions. The two-stage strategy—benchmark-referenced operating points for model evaluation and a separate, prospectively fixed operating point for biological inference—reflects standard practice in diagnostic machine learning, where operating-point selection depends on downstream application constraints; we return to the implications of threshold transferability across distributions in the Discussion.

**Table 3:** Evaluation metrics on the independent test dataset (n=110; 33 amyloidogenic and 77 non-amyloidogenic sequences).^a^

Model	Accuracy	Sensitivity	Specificity	MCC	AUC
VAEBAC (τ=0.43919, Youden J)	0.845	**0.848**	0.844	0.660	0.904
VAEBAC (τ=0.9354, benchmark-calibrated)	0.891	0.697	**0.974**	0.734	0.904
CANYA	0.590	0.360	0.796	0.170	0.520
AMYPred-FRL	0.873	**0.848**	0.883	0.710	0.902
ET-CTDD	0.855	0.788	0.883	0.660	**0.905**
LR-APAAC	0.773	0.697	0.805	0.484	0.832
ET-APAAC	0.818	0.606	0.909	0.549	0.860
LR-DDE	0.809	0.788	0.818	0.577	0.885
RF-CTDD	0.836	0.788	0.857	0.586	0.897

a VAEBAC is reported at two operating thresholds to illustrate the dependence of threshold-based metrics on the selected decision boundary. The Youden J-optimal threshold (τ=0.43919) was determined by maximizing Youden’s J statistic on the independent test dataset. The second threshold (τ=0.9354) represents a high-specificity operating point obtained by calibration on the same independent test dataset. Because this threshold was selected using the benchmark labels, its threshold-dependent metrics are post hoc and should not be interpreted as unbiased estimates of out-of-sample performance. AUC is calculated independently from the continuous prediction scores and is therefore threshold-independent.

To assess whether the nominal 512-dimensional latent space was necessary for classification performance, we conducted a post-hoc dimensionality sufficiency analysis using a lightweight probe classifier trained via five-fold cross-validation on PCA-reduced projections of the latent representation, and separately on pre-bottleneck pooled features bypassing the VAE encoder entirely (Table 9, available as supplementary data at *Bioinformatics* online).

For CANYA (Thompson *et al.* 2025), predictions were generated using the official implementation available from the authors’ GitHub repository. The classification threshold for reporting accuracy-based metrics was determined using Youden’s J statistic on the validation set.

For comparison with protein language model (PLM)-based approaches, we used the identical training dataset provided in the FA-Amy GitHub repository to ensure direct methodological comparability. Protein embeddings were generated using the ESMC 600M model (esmc_600m_2024_12_v0) obtained from the EvolutionaryScale ESM C release (Candido *et al.* 2026). All embeddings were produced using the same model checkpoint and preprocessing pipeline as described in the FA-Amy implementation. Downstream training was performed on the same benchmark dataset used in FA-Amy without modification.

To evaluate cross-dataset generalizability, we evaluated VAEBAC on an external dataset constructed by Yang *et al.* (2023). The dataset was not used for model training or selection of the benchmark-calibrated threshold, and was evaluated only after the model and operating threshold had been fixed. This dataset was used to assess the robustness of both PLM-based models and VAEBAC under distributional shift.

For comparability with the FA-Amy evaluation protocol, the cross-dataset FA-Amy comparison (Results; Table 6, available as supplementary data at *Bioinformatics* online) applied that protocol’s input handling: sequences were truncated to their first 1000 residues prior to evaluation, and any residue outside the model’s 23-character alphabet was recoded as X. All 1107 sequences of the Yang *et al.* dataset were retained under this protocol; truncation affected 338 sequences (30.5%; 7 positive; 331 negative) and recoding affected 1 sequence. This input handling differs from that used for the primary external evaluation reported above, in which sequences exceeding the model’s 10 746-residue maximum (6 negatives) and one selenocysteine-containing negative were instead excluded, leaving *n* = 1100. The two external analyses are therefore reported separately throughout and should not be pooled or compared directly.

To examine potential redundancy and mitigate homology-induced performance inflation, sequence clustering was performed using CD-HIT v4.8.1 (Fu *et al.* 2012) at a 40% sequence identity threshold. This homology-aware analysis ensured that closely related sequences were not inadvertently distributed across training and evaluation partitions. This analysis used a separately trained checkpoint sharing identical frozen encoder weights with the primary reported model, retrained from initialization at each threshold to ensure a controlled, consistent comparison (see Table 7, available as supplementary data at *Bioinformatics* online, for details).

### 2.4 Sequence length analysis

To assess whether sequence length constitutes a confounding predictive signal, we compared length distributions between amyloidogenic and non-amyloidogenic classes in the training, independent, and external (Yang *et al.*) test sets using the Mann-Whitney *U* test, with effect size reported as Cohen’s *d*. We additionally trained a logistic regression classifier using sequence length as the sole predictor, evaluated via repeated (20×) five-fold stratified cross-validation on the independent and external test sets, and assessed transfer of this signal by evaluating a length-only classifier fitted on the training set directly on the external test set. Statistical significance of the AUC difference between VAEBAC and the length-only baseline was assessed via DeLong’s test (DeLong *et al.* 1988). As a further validity check, we performed a label-permutation test: training labels were randomly shuffled (preserving class balance), and the classifier head was retrained from initialization (encoder frozen) and evaluated by five-fold cross-validation. We further compared a joint logistic regression model (length and VAEBAC score) against a length-only model via a likelihood-ratio test.

To characterize the length dependence of the classifier head specifically, rather than of the latent space as a whole, we computed SHAP attributions for the classifier head over the frozen 512-dimensional latent representation of the 110-sequence independent benchmark, using DeepExplainer with the 318 training-set latent vectors as the background distribution. For every latent dimension we then computed the Pearson correlation between its value across the benchmark and the corresponding true (unpadded) sequence length, with *P*-values Bonferroni-corrected across all 512 dimensions. Finally, we evaluated performance within sequence-length strata: the independent benchmark was partitioned into quartiles by true sequence length, and accuracy, MCC and AUC were computed within each quartile at the fixed benchmark-calibrated threshold (τ=0.9354), with 95% confidence intervals for MCC obtained by bootstrap resampling (1000 resamples). Quartiles containing fewer than five positive sequences are reported by AUC alone, as MCC is unstable at that sample size. All analyses in this paragraph used the primary reported checkpoint with the encoder and classifier head frozen; no component was retrained.

### 2.5 Evaluation metrics

Model performance was evaluated using multiple complementary metrics to comprehensively assess classification accuracy, robustness, and correlation between predicted and true labels. For binary classification, accuracy, precision, recall, F1-score, Matthews correlation coefficient (MCC), and area under the receiver operating characteristic curve (AUC–ROC) were computed. In addition, Pearson correlation coefficient was used to quantify the linear agreement between predicted probabilities and ground-truth labels.

Let TP, TN, FP, and FN denote the number of true positives, true negatives, false positives, and false negatives, respectively. The evaluation metrics were defined as follows:


(1) $$\text{Accuracy}=\frac{TP+TN}{TP+TN+FP+FN},$$

(2) $$\text{MCC}=\frac{\left(TP\times TN)-(FP\times FN\right)}{\sqrt{\left(TP+FP)(TP+FN)(TN+FP)(TN+FN\right)}}.$$

The MCC metric was included due to its robustness to class imbalance and its ability to provide a balanced assessment of binary classifiers.


(3) $$\text{TPR}=\frac{TP}{TP+FN},\;\text{FPR}=\frac{FP}{FP+TN}.$$

The area under the ROC curve (AUC–ROC) was used as a threshold-independent measure of classification performance.


(4) $$r=\frac{\sum_{i=1}^{n}\left(y_{i}-\bar{y}\right)({\hat{y}}_{i}-\bar{\hat{y}})}{\sqrt{\sum_{i=1}^{n}{\left(y_{i}-\bar{y}\right)}^{2}}\sqrt{\sum_{i=1}^{n}{\left({\hat{y}}_{i}-\bar{\hat{y}}\right)}^{2}}},$$

where $y_{i}$ and ${\hat{y}}_{i}$ denote the true labels and predicted probabilities for sample *i*, respectively.

### 2.6 Proteome-scale screening and candidate selection

Proteome-wide screening was performed using reviewed (Swiss-Prot) *Escherichia coli* K-12 entries retrieved from UniProt (UniProt Consortium 2023) (taxonomy_id: 83333 which includes substrains DH10B and MC4100/BW2952; 6066 entries). Entries longer than 100 residues were removed to facilitate downstream experimental characterization, leaving 709 sequences, which were submitted to VAEBAC. Note that this entry set is broader than the one used for the nucleic-acid-binding protein retrieval described below, which was restricted to the parent taxonomic node and therefore excludes substrain entries.

For each protein, the model produced a scalar output in the range 0–1 after sigmoid activation, which was used as the predicted probability for amyloidogenicity. Proteins were classified as amyloidogenic candidates using a stringent probability threshold of 0.95, rather than the default 0.5, to prioritize high-confidence predictions. From this filtered set, the top three candidate proteins (UniProt IDs: P0AA04, P0C037, and B1XF10) with the highest predicted probabilities were selected for further experimental validation.

Codon-optimized gene constructs encoding each of the selected proteins were synthesized and cloned into bacterial expression vectors by GenScript. The resulting plasmids were transformed into *Escherichia coli* BL21 (DE3) competent cells for heterologous expression.

Transformed cells were grown in Luria–Bertani (LB) medium supplemented with ampicillin at 37 °C until mid-log phase (${\text{OD}}_{600}\approx 0.6$–0.8). Protein expression was induced by the addition of isopropyl β-D-1-thiogalactopyranoside (IPTG) to a final concentration of 0.2 mM. Following induction, cultures expressing protein P0AA04 were incubated at 37 °C for 6 h, whereas cultures expressing proteins P0C037 and B1XF10 were incubated at 16 °C for 8 h to reduce aggregation and inclusion body formation.

Cells were harvested by centrifugation and lysed using standard lysis procedures. Recombinant proteins were purified under native conditions using nickel–nitrilotriacetic acid (Ni–NTA) affinity chromatography. Protein purity and molecular weight were confirmed by SDS–PAGE; representative expression induction and purification gels are provided in Fig. 2, available as supplementary data at *Bioinformatics* online.

**Figure 2:**
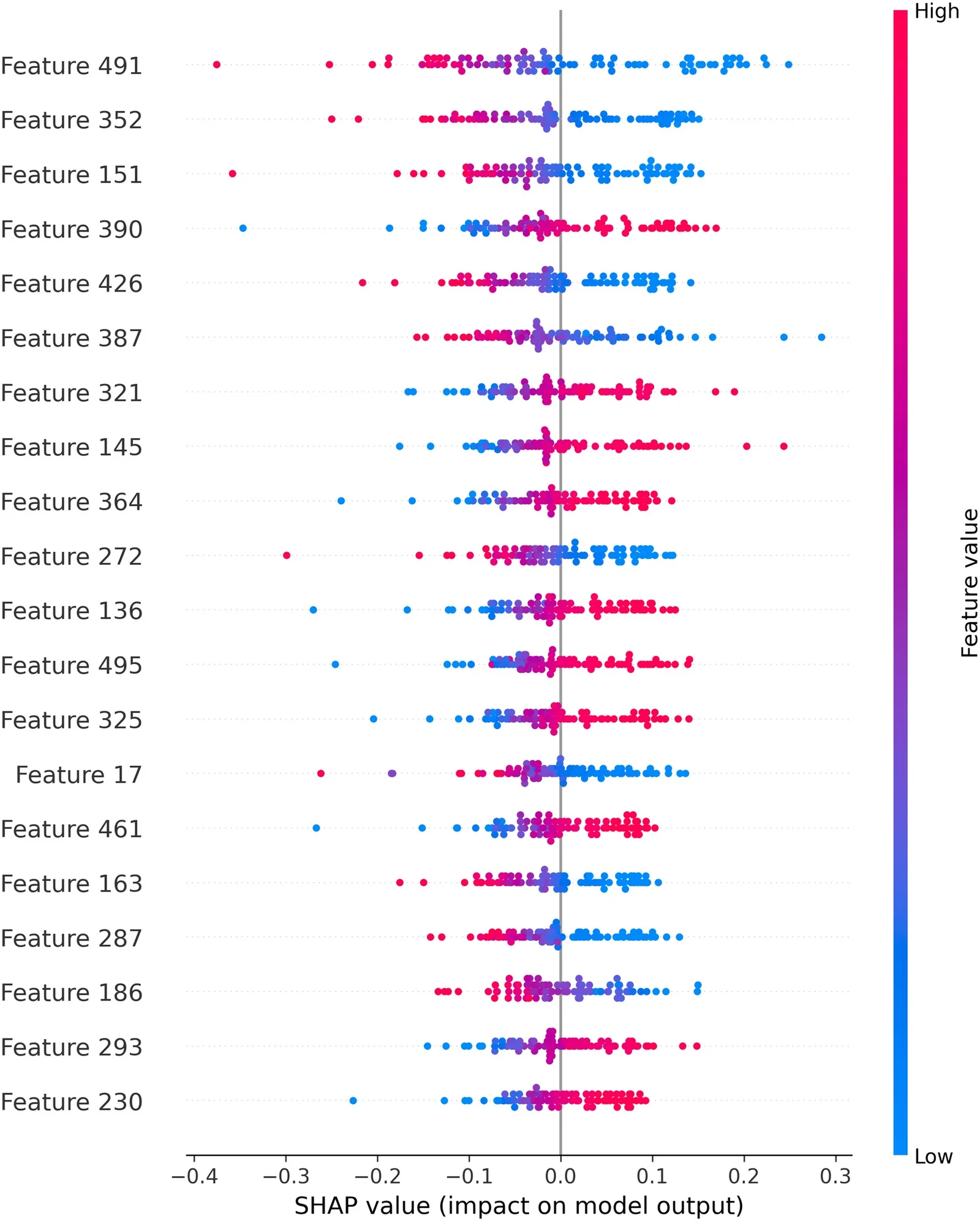
SHAP summary plot illustrating the contribution of latent features to VAEBAC predictions. Each point represents the SHAP value of a feature for a single protein sequence in the test dataset. Positive SHAP values indicate features contributing toward amyloidogenic classification, whereas negative values indicate contributions toward the non-amyloid class. Multiple latent dimensions contribute to individual predictions; the highest-contributing dimensions are strongly correlated with sequence length (see Section 3.2).

### 2.7 *In vitro* aggregation and biophysical characterization

Purified recombinant proteins were subjected to *in vitro* aggregation assays under identical conditions. Proteins were diluted to a final concentration of 5 μM and incubated at 37 °C with constant agitation (350 rpm) using a thermomixer. Aggregation kinetics were monitored by Thioflavin T (ThT) fluorescence spectroscopy and circular dichroism at the time points indicated; representative time-resolved CD spectra for each protein are provided in Fig. 3, available as supplementary data at *Bioinformatics* online.

**Figure 3:**
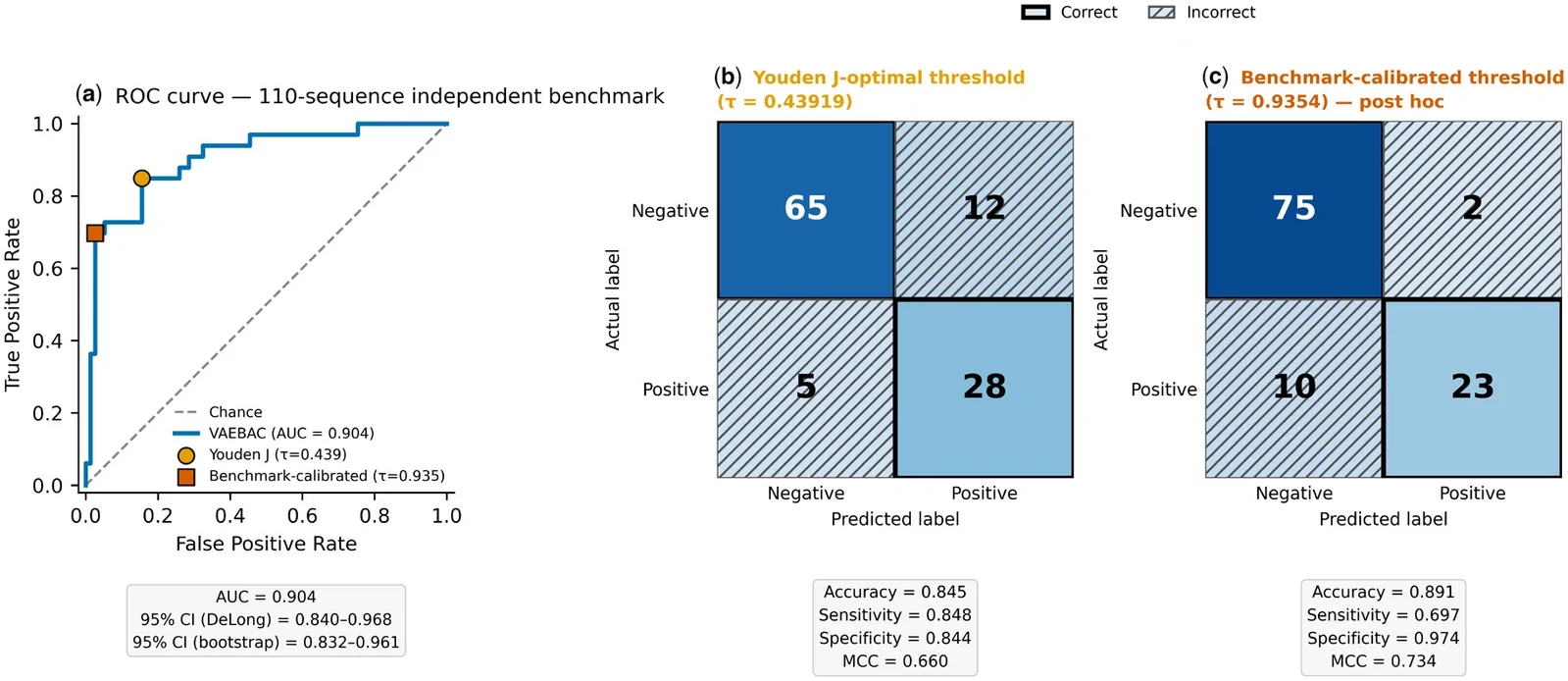
Performance of VAEBAC on the complete 110-sequence independent benchmark (33 amyloidogenic, 77 non-amyloidogenic). (a) ROC curve illustrating discrimination between amyloidogenic and non-amyloidogenic proteins (AUC = 0.904; DeLong 95% CI, 0.840–0.968; bootstrap 95% CI, 0.832–0.961). The ROC curve is threshold-independent; because VAEBAC outputs a continuous score, many distinct confusion matrices can be generated from it depending on the operating threshold chosen, as illustrated by the two panels below. (b) Confusion matrix at the Youden J-optimal threshold (τ=0.43919), a balanced operating point (accuracy 0.845, sensitivity 0.848, specificity 0.844, MCC 0.660). (c) Confusion matrix at the benchmark-calibrated threshold (τ=0.9354; accuracy 0.891, sensitivity 0.697, specificity 0.974, MCC 0.734). This threshold was obtained by scanning the same benchmark shown here and is therefore a post hoc, high-specificity operating point rather than an independently predetermined one; it is shown for transparency and because it was the operating point subsequently applied unchanged to the external Yang *et al.* dataset (see Results). A prevalence-adjusted random baseline and majority-class comparison are additionally reported in Fig. 13 and Table 11, available as supplementary data at *Bioinformatics* online.

Secondary structure changes during aggregation were assessed using circular dichroism (CD) spectroscopy (JASCO-J815 spectropolarimeter). Thioflavin T (ThT) binding assays were performed by incubating protein samples with ThT at a 1:2 protein-to-dye ratio in the dark for 20 min, followed by fluorescence emission measurements between 400 and 600 nm using a Shimadzu fluorescence spectrophotometer (RF-6000). For atomic force microscopy (AFM) imaging, 10 μl of protein was deposited onto freshly cleaved mica, incubated for 2–5 min, gently rinsed with Milli-Q water, and air-dried at room temperature.

### 2.8 Identification of nucleic-acid-binding proteins across organisms

Reviewed proteome entries for each organism were retrieved from UniProt using the nucleic-acid-binding molecular function annotation (GO:0003676, any evidence code). Searches were performed using the following taxon identifiers, restricted in each case to the parent taxonomic node so that substrain-level entries were excluded: *E. coli* K-12 (taxon ID: 83 333), *B. subtilis* 168 (taxon ID: 2 24 308), *S. cerevisiae* S288C (taxon ID: 5 59 292), and *H. sapiens* (taxon ID: 9606). Only Swiss-Prot reviewed entries were retained to ensure annotation quality. This procedure yielded 772, 568, 1264, and 4005 nucleic-acid-binding proteins for *E. coli*, *B. subtilis*, *S. cerevisiae*, and *H. sapiens* respectively.

### 2.9 Whole-protein amyloidogenicity prediction using VAEBAC

Amyloidogenic propensity was predicted at the whole-protein level using VAEBAC, a deep generative model trained to classify proteins as amyloidogenic or non-amyloidogenic based on global sequence features. All NABPs in each organism were submitted to VAEBAC under identical inference parameters. A uniform classification threshold of 0.95 was applied across all four organisms to ensure methodological consistency and to avoid dataset-specific bias. Use of this stringent threshold results in a conservative set of amyloidogenic NABPs, reducing potential false-positive inflation in downstream functional enrichment analyses. The numbers of amyloidogenic and non-amyloidogenic NABPs identified in each organism are summarized in Table 1.

### 2.10 Gene ontology enrichment analysis

Functional enrichment analysis was performed separately for amyloidogenic and non-amyloidogenic NABP subsets in each organism using the Gene Ontology knowledgebase with the PANTHER Classification System (http://pantherdb.org/) as the annotation tool (Ashburner *et al.* 2000, Thomas *et al.* 2022, Gene Ontology Consortium 2026). Overrepresentation tests were conducted against the respective organism’s complete reference proteome using Fisher’s exact test with false discovery rate (FDR) correction for multiple testing. Enrichment significance was assessed using adjusted *P*-values (FDR < 0.05). To assess independence from GO annotation source, enrichment results for *H. sapiens* were independently validated using g: Profiler (https://biit.cs.ut.tartu.edu/gprofiler/) (Kolberg *et al.* 2023). Additionally, REVIGO semantic clustering (Supek *et al.* 2011) was applied to *H. sapiens* GO enrichment results to reduce annotation redundancy and confirm that the observed enrichment reflects non-redundant biological processes. Importantly, results from all three tools were consistent, confirming that the reported enrichment pattern is independent of the annotation framework employed.

### 2.11 Fisher’s exact test for transcription factor enrichment

To provide a direct hypothesis-driven statistical assessment of transcription factor enrichment among amyloidogenic NABPs, independent of GO enrichment pipelines, Fisher’s exact test was performed for eukaryotic organisms. Proteins annotated with GO:0003700 (DNA-binding transcription factor activity) were retrieved from UniProt for *S. cerevisiae* and *H. sapiens* and classified as transcription regulators. A 2×2 contingency table was constructed for each organism with amyloidogenic/non-amyloidogenic NABP classification as rows and transcription regulator/non-transcription regulator annotation as columns. One-sided Fisher’s exact test was performed using the scipy.stats.fisher_exact function in Python (Virtanen *et al.* 2020). This annotation-dependent analysis was restricted to eukaryotic organisms where transcription factor GO annotation is most complete; prokaryotic transcriptional regulation relies on mechanistically diverse sigma factor-mediated and two-component regulatory systems that are incompletely captured by GO:0003700 annotations, limiting the interpretive power of annotation-dependent tests for bacterial proteomes.

### 2.12 Visualization

All figures were generated using Matplotlib and Seaborn in Python. GO enrichment bar charts and heatmaps were generated using custom Python scripts. Fisher’s exact test stacked bar charts and contingency count plots were produced using Matplotlib.

### 2.13 Web server implementation

A publicly accessible web server was developed at https://www.vaebac.com. The server enables whole-protein amyloidogenicity prediction from FASTA sequences in single or batch mode. For each input sequence, it reports the predicted probability score, binary classification outcome, and confidence metrics. The server was implemented using HTML, CSS, and JavaScript for the front-end, with a Python Flask framework handling back-end model inference.

## 3 Results

### 3.1 Model performance and generalization

The model was evaluated using stratified five-fold and 10-fold cross-validation on the training dataset (Table 2). The model reported an accuracy of 0.875±0.021 and MCC of 0.713±0.054 under five-fold cross-validation and 0.878±0.019 accuracy with MCC of 0.721±0.043 under ten-fold cross-validation. AUC values were high (∼0.94) under both settings, indicating strong class discrimination within the cross-validation framework.

On the complete 110-sequence independent benchmark (33 amyloidogenic, 77 non-amyloidogenic), VAEBAC showed competitive performance relative to established predictors, with an AUC of 0.904 (Table 3). The AUC values of the leading methods were closely clustered (VAEBAC 0.904; ET-CTDD 0.905; AMYPred-FRL 0.902), so we do not interpret these small differences as evidence that any single method dominates on threshold-independent discrimination. At the benchmark-calibrated operating point (τ=0.9354; see Methods), VAEBAC achieved high specificity (0.974) and MCC (0.734), while AMYPred-FRL achieved higher sensitivity (0.848 versus 0.697); at the Youden J-optimal operating point (τ=0.43919) VAEBAC’s sensitivity and specificity were more balanced (0.848 and 0.844, respectively). Because the benchmark-calibrated threshold was obtained by scanning the same benchmark it is evaluated on, its accuracy, sensitivity, specificity and MCC values are post hoc and are reported for transparency rather than as evidence of superior generalization. SHAP analysis (Lundberg and Lee 2017) (Fig. 2) identified the latent dimensions most influential to classifier predictions; we characterize the biological/representational basis of these dimensions below (see Discussion). The classification behaviour of VAEBAC on the independent test set is further illustrated by the ROC curve and the two corresponding confusion matrices (Fig. 3). To assess whether reported cross-validation performance was influenced by sequence redundancy, we repeated five-fold cross-validation after CD-HIT clustering at four identity thresholds (40–90%). Performance remained stable across all thresholds (Accuracy: 0.868–0.887; MCC: 0.700–0.739; AUC: 0.936–0.941), closely matching the unfiltered baseline, indicating that reported performance is not attributable to redundancy-driven fold leakage (Table 7, available as supplementary data at *Bioinformatics* online). A classifier trained on AAC-PSSM features underperformed the one-hot/physicochemical representation across all metrics (five-fold CV AUC: 0.829 versus 0.944; independent test AUC: 0.830 versus 0.904; Table 8, available as supplementary data at *Bioinformatics* online), indicating the chosen representation provides superior discriminative performance on this task.

### 3.2 Latent representation reflects a length-correlated signal

Principal component analysis of the 512-dimensional latent space revealed that the dominant component (94.8% of variance) correlated strongly with input sequence length (Pearson r=0.990; 110-sequence independent benchmark; primary reported checkpoint, raw_model_1.pth). Correlation analysis of the top 15 SHAP-contributing latent dimensions (Fig. 2) similarly showed strong association with sequence length (Spearman |ρ|=0.53–0.98), with weaker secondary correlations to compositional features. This SHAP analysis, and Fig. 2, were computed on the 87-sequence version of the independent test set using a separately trained checkpoint (deposited on Zenodo) that shares the pretrained encoder with the primary reported model; attributions were obtained with shap. GradientExplainer applied to the classifier head over the latent μ representation. Class-wise sequence lengths differed substantially across all three evaluated datasets (training, independent, and external test sets; Table 10, available as supplementary data at *Bioinformatics* online). A logistic regression classifier using sequence length alone achieved an AUC of 0.789±0.110 on the complete 110-sequence independent benchmark (repeated 20× five-fold stratified cross-validation) and 0.828±0.036 on the external (Yang *et al.*) test set, compared with 0.904 and 0.929 for the full VAEBAC model, respectively. Of the 1107 raw Yang *et al.* sequences (161 positive, 946 negative), 1 negative was excluded because it contained selenocysteine (outside the model’s amino-acid alphabet) and 6 negatives exceeded the model’s maximum input length, leaving *n* = 1100 (161 positive, 939 negative) for this analysis. A length-only classifier trained on the training set and evaluated directly on the external test set achieved an AUC of 0.828, closely matching its in-distribution performance, indicating that this signal transfers across independently constructed benchmark datasets. The difference in discriminative performance between the full VAEBAC model and the length-only baseline was statistically significant on both the independent benchmark (DeLong’s test, z=2.775, p=0.0055) and the external test set (DeLong’s test, z=5.71, $p=1.2\times 10^{-8}$), confirming that VAEBAC captures substantial predictive signal beyond sequence length alone. As a complementary, full-sample analysis, a logistic regression model predicting the true label from sequence length and VAEBAC’s continuous score jointly significantly outperformed a length-only model on both the independent benchmark (likelihood-ratio test, LR=37.32, $p=1.0\times 10^{-9}$) and the external test set (LR=255.37, $p=1.8\times 10^{-57}$; both df=1), further confirming that VAEBAC captures predictive signal beyond sequence length alone.

We extended this analysis to the classifier head itself, to test whether the head learns to discount the length-encoding latent dimensions. It does not. Of the 20 latent dimensions ranked highest by mean |SHAP value| on the independent benchmark, 19 correlate with true sequence length at |r|>0.3 (median |r|=0.91, maximum |r|=0.99; all Bonferroni-corrected *P* <.01 across 512 dimensions), and across the full latent space the median is |r|=0.96 (Table 12, Figs 15 and 16, available as supplementary data at *Bioinformatics* online). The latent principal component analysis reported above is consistent across datasets: the corresponding values are 93.8% of variance and r=0.977 on the 318-sequence training set, and 90.9% and r=0.965 on the 9180-sequence AmyPDB pretraining corpus. The dimensions the classifier relies upon most heavily are therefore precisely the length-correlated ones, consistent with the pooling mechanism described in Methods.

Performance nonetheless persists within restricted length ranges (Table 13, Figs 17 and 18, available as supplementary data at *Bioinformatics* online). Stratifying the independent benchmark into sequence-length quartiles at the fixed benchmark-calibrated threshold, VAEBAC achieved AUC = 0.917 and MCC = 0.801 (95% CI, 0.596–1.000) in Q1 (11–157 residues, n=28), AUC = 0.809 and MCC = 0.574 (0.189–0.892) in Q2 (168–451 residues, n=27), AUC = 0.902 in Q3 (459–972 residues, n=27), and AUC = 0.731 in Q4 (1070–10 746 residues, n=28). Q3 and Q4 contain only four and two positive sequences respectively, so MCC is not reported for those strata. Discrimination remains above chance in every quartile, but it is not uniform: AUC is lowest in the longest quartile, which is the pattern expected if part of the signal is length-driven. We therefore present this stratification as evidence that length-independent signal is present throughout the length range, not as evidence of length-invariance.

Finally, we tested the length dependence causally by removing the leakage channel directly. Replacing the encoder’s adaptive average pooling with masked mean pooling—averaging only over each sequence’s true unpadded positions rather than across the full padded width of 10 746—eliminates the length signal almost entirely, with no retraining of any weight. Under masked pooling the correlation between the dominant latent principal component and sequence length falls from |r|=0.990 to |r|=0.025; the median absolute correlation of the 20 highest-SHAP latent dimensions with length falls from 0.908 to 0.028; and whereas 19 of those 20 dimensions were significantly length-correlated under the original pooling, none remains significant after Bonferroni correction. Discrimination is largely preserved: AUC falls from 0.904 to 0.868, a decrease of 0.036, while Matthews correlation coefficient at each operating point’s Youden-optimal threshold *improves* from 0.660 to 0.687 and accuracy from 0.845 to 0.873 (Fig. 19, available as supplementary data at *Bioinformatics* online). Because the encoder and classifier head were fitted under the original pooling scale and were left frozen here, this comparison is an inference-time substitution rather than a like-for-like model, and the retained performance should be read as a lower bound on what an equivalently retrained model would achieve. We note one important caveat: masked pooling shifts the latent scale the classifier head was calibrated under, so the resulting probabilities are not calibrated—all fall below 0.5—and only the ranking and the Youden-optimal operating point are interpretable. The absolute scores, and the benchmark-calibrated threshold (τ=0.9354), do not transfer to the masked-pooling setting.

### 3.3 Performance on the external Yang *et al.* dataset

To assess generalization beyond the 110-sequence independent benchmark, we evaluated VAEBAC on the external dataset constructed by Yang *et al.* (2023) (Methods), applying the benchmark-calibrated threshold (τ=0.9354) unmodified, without any re-optimization on Yang *et al.* labels. Of 1107 raw sequences (161 positive, 946 negative), seven negatives were excluded during preprocessing (one containing selenocysteine, outside the model’s amino-acid alphabet; six exceeding the model’s maximum input length), leaving a full evaluated set of n=1100 (161 positive, 939 negative). On this full set, VAEBAC achieved AUC = 0.929 (DeLong 95% CI, 0.906–0.952; bootstrap 95% CI, 0.905–0.952), accuracy = 0.938, and MCC = 0.740 (TN = 916, FP = 23, FN = 45, TP = 116).

However, sequence-level comparison against the development data revealed that, of the 161 Yang *et al.* positive sequences, 27 overlap the 110-sequence benchmark and 109 overlap the training pool (136 in total), with the remaining 25 novel relative to both; overlap is therefore concentrated primarily in the training pool rather than in the benchmark used for threshold calibration. Consequently, the full-set result above should not be interpreted as a fully independent validation, although the τ=0.9354 threshold itself was selected prior to, and was not re-optimized on, the Yang *et al.* data. We therefore also report performance on the strictly novel subset: sequences absent from both the training data and the independent benchmark (n=961; 25 positive; 936 negative). On this subset, at the same fixed threshold, VAEBAC achieved AUC = 0.906 (DeLong 95% CI, 0.831–0.981; bootstrap 95% CI, 0.823–0.973), accuracy = 0.970, sensitivity = 0.760, specificity = 0.975, and MCC = 0.573 (TN = 913, FP = 23, FN = 6, TP = 19).

The discrimination (AUC) achieved on the strictly novel subset (0.906) is comparable to that on the 110-sequence benchmark itself (0.904), suggesting that VAEBAC’s continuous ranking of sequences by amyloidogenicity is reasonably stable under this distribution shift. In contrast, MCC at the fixed operating threshold declines from 0.740 (full, overlapping set) to 0.573 (novel-only), indicating that threshold-dependent binary calibration is less stable than threshold-independent discrimination across datasets. We emphasize that the novel-only subset contains only 25 positive sequences, so its MCC and sensitivity estimates carry substantial statistical uncertainty and should not be over-interpreted as a precise point estimate; MCC itself remains an appropriate metric under class imbalance; the limiting factor here is the small absolute number of novel positive examples rather than the class ratio. Taken together, these results indicate that users applying VAEBAC to sequence distributions substantially different from the training/benchmark data should consider re-calibrating the operating threshold rather than assuming the benchmark-calibrated value transfers unchanged.

### 3.4 External validation reveals differential performance retention

Using the benchmark dataset and training protocol provided in the FA-Amy repository, we conducted an independent cross-dataset evaluation of FA-Amy and VAEBAC (Table 6, available as supplementary data at *Bioinformatics* online). This analysis follows FA-Amy’s benchmark and evaluation protocol and is reported separately from the Yang *et al.* full- and novel-only analyses described above. In this analysis, FA-Amy achieved higher internal cross-validation performance (MCC = 0.842) than VAEBAC (MCC = 0.730); this VAEBAC value is distinct from, though numerically close to, the 110-sequence benchmark-calibrated MCC of 0.734 reported in Table 3, as it derives from a separately conducted internal evaluation. Under external evaluation, FA-Amy’s MCC decreased to 0.492 (58.4% retention relative to its own internal performance), whereas VAEBAC’s external MCC of 0.657 corresponds to 90.0% retention relative to its internal performance. In this independent cross-dataset evaluation, VAEBAC therefore retained a substantially greater fraction of its internal MCC than FA-Amy (90.0% versus 58.4%), despite FA-Amy having the higher internal MCC. We report retention here as secondary, descriptive evidence rather than a primary claim of overall superiority; the AUC-based comparison on the strictly novel Yang subset described above is the more direct threshold-independent assessment of generalization. Although AUROC values were comparable (FA-Amy: 0.918; VAEBAC: 0.914), threshold-dependent metrics differed: VAEBAC showed higher external balanced accuracy (0.851 versus 0.832), higher precision (0.656 versus 0.384), and higher F1-score (0.709 versus 0.540) than FA-Amy under this cross-dataset evaluation. To properly characterize random-classifier behavior under class imbalance, we additionally evaluated a majority-class baseline (70.0% accuracy, MCC = 0, illustrating that accuracy alone is misleading under imbalance) and a prevalence-weighted random baseline (10 000 trials; mean AUC = 0.501±0.060, MCC = 0.000±0.096), against which VAEBAC’s performance is substantially and consistently superior. A label-permutation test, in which VAEBAC’s own training procedure was applied to randomly shuffled labels, yielded near-chance-level performance (Accuracy 0.582±0.127; MCC 0.039±0.283, consistent with zero within one standard deviation; AUC 0.537±0.169), confirming that reported performance reflects genuine learned association between sequence and label rather than an artifact of model architecture or training procedure (Fig. 13, Table 11, available as supplementary data at *Bioinformatics* online).

### 3.5 Homology-controlled evaluation confirms robustness

As a further, separate robustness check, sequence clustering was performed using CD-HIT at 40% sequence identity, and training/test partitions were reconstructed to eliminate homologous overlap between training and evaluation data. This homology-controlled evaluation is distinct from the cross-dataset comparison described above and from the Yang *et al.* analysis. Under this homology-controlled setting, FA-Amy achieved sensitivity = 0.9130, specificity = 0.8457, accuracy = 0.8555, balanced accuracy = 0.8794, MCC = 0.6063, AUC = 0.9394, and F1-score = 0.6476, whereas VAEBAC achieved an external MCC of 0.744. Because MCC was the directly evaluated metric available for VAEBAC under this specific homology-controlled split, we restrict the comparison to MCC and do not infer corresponding balanced accuracy or F1-score values for VAEBAC. Since directly comparable internal MCC values were not available for both models under this specific homology-controlled protocol, no retention percentage is reported; the absolute external metrics are presented separately.

### 3.6 Experimental validation

To independently validate model predictions, three high-confidence amyloidogenic candidates (P0AA04, P0C037, and B1XF10) were experimentally confirmed using Thioflavin T (ThT) fluorescence spectroscopy (Fig. 4a–c). The ThT fluorescence intensity of all three proteins increased with time, consistent with β-sheet-rich amyloid aggregate formation. Throughout the 96-h observation period, P0AA04 showed a steady gradual increase, suggesting slow but continuous fibril formation. P0C037 rapidly reached near-maximum fluorescence in approximately 2 h, consistent with rapid nucleation and fibril development. B1XF10 showed an intermediate profile, with a clear fluorescence rise after approximately 10 h followed by a slower aggregation phase lasting up to 24 h. AFM imaging (Fig. 4d–f) confirmed that all three proteins formed abundant long unbranched fibrillar structures with nanometre-scale height distributions, consistent with mature amyloid morphology.

**Figure 4:**
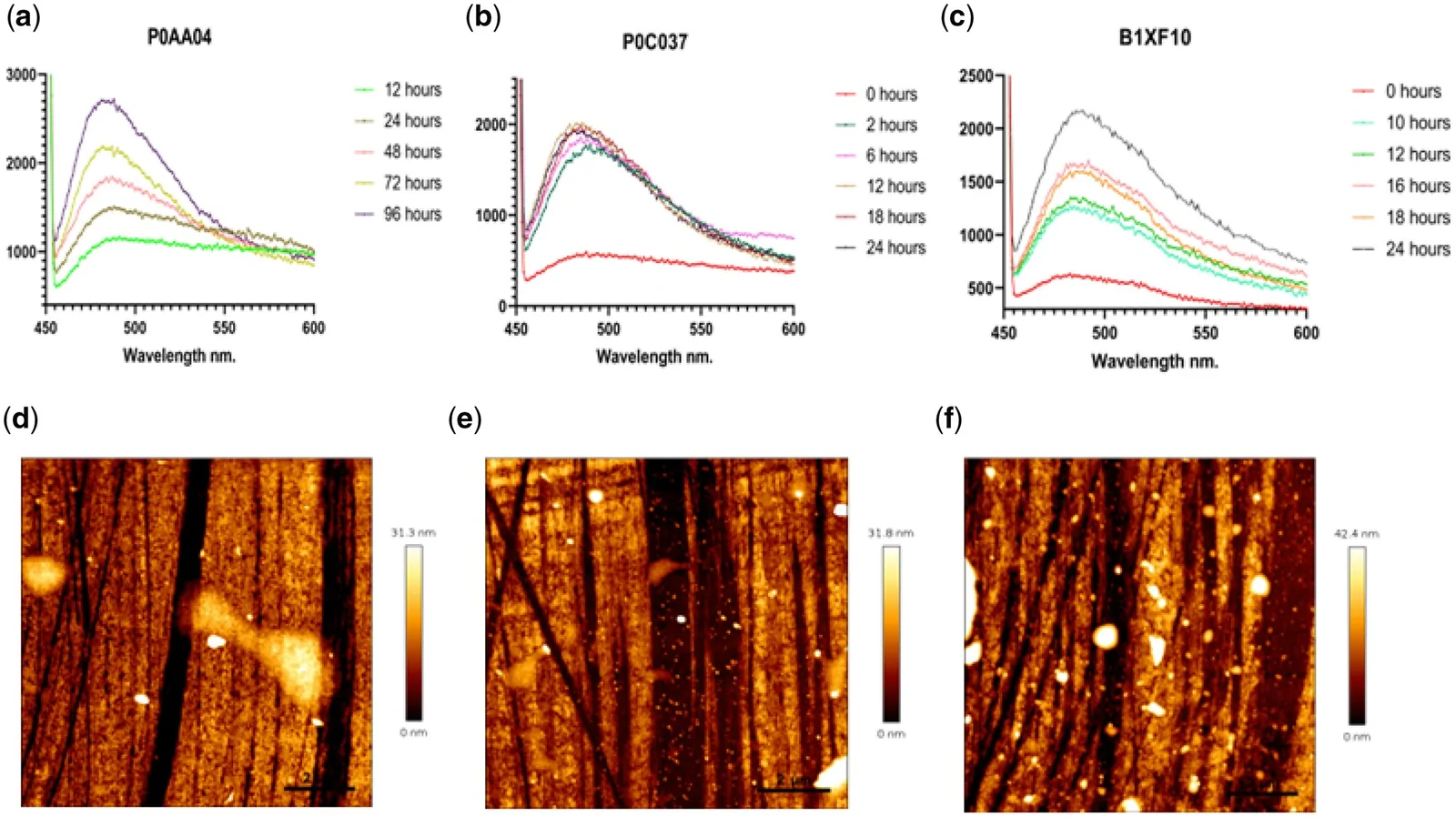
Experimental validation of VAEBAC-predicted amyloidogenic proteins. (a–c) Thioflavin T fluorescence aggregation kinetics for proteins P0AA04, P0C037, and B1XF10, respectively. Increased fluorescence intensity indicates formation of β-sheet-rich amyloid aggregates over time. (d–f) Atomic force microscopy images showing fibrillar amyloid structures formed by the respective proteins after incubation.

### 3.7 Cross-organism conservation of functional stratification in amyloidogenic NABPs

To examine whether the functional distribution of amyloidogenic NABPs is conserved across phylogenetically diverse organisms, VAEBAC was applied to the complete nucleic-acid-binding proteomes of *E. coli*, *B. subtilis*, *S. cerevisiae*, and *H. sapiens* under a uniform classification threshold. The numbers of predicted amyloidogenic NABPs identified in each organism are provided in Table 1.

GO overrepresentation analysis revealed a consistent pattern of transcriptional regulatory enrichment across all four organisms (Fig. 5). Importantly, this pattern was not dependent on a single annotation framework: consistent results were obtained across PANTHER, g: Profiler, and REVIGO semantic clustering, confirming annotation-tool independence of the findings. In *E. coli*, amyloidogenic NABPs were significantly enriched within categories including regulation of DNA-templated transcription (fold enrichment = 9.31; FDR = 4.39 × $10^{-2}$), regulation of RNA metabolic process (fold enrichment = 9.06; FDR = 3.36 × $10^{-2}$), and regulation of gene expression (fold enrichment = 7.86; FDR = 4.07 × $10^{-2}$). An equivalent pattern was observed in *B. subtilis*, where amyloidogenic NABPs were enriched in regulation of RNA metabolic process (fold enrichment = 9.53; FDR = 4.99 × $10^{-4}$) and regulation of gene expression (fold enrichment = 8.95; FDR = 2.04 × $10^{-4}$). In *S. cerevisiae*, significant enrichment was observed for regulation of transcription by RNA polymerase II (fold enrichment = 5.00; FDR = 1.31 × $10^{-4}$) and negative regulation of transcription by RNA polymerase II (fold enrichment = 10.72; FDR = 6.69 × $10^{-5}$). In *H. sapiens*, amyloidogenic NABPs were significantly enriched in regulation of transcription by RNA polymerase II (fold enrichment = 4.25; FDR = 1.47 × $10^{-39}$), regulation of RNA metabolic process (fold enrichment = 3.78; FDR = 9.23 × $10^{-48}$) and regulation of gene expression (fold enrichment = 2.95; FDR = 4.12 × $10^{-39}$). Conversely, non-amyloidogenic NABPs were consistently enriched across constitutive biosynthetic and RNA-processing functions in all four organisms, including ribosome biogenesis, tRNA processing, and constitutive RNA metabolic processes.

**Figure 5:**
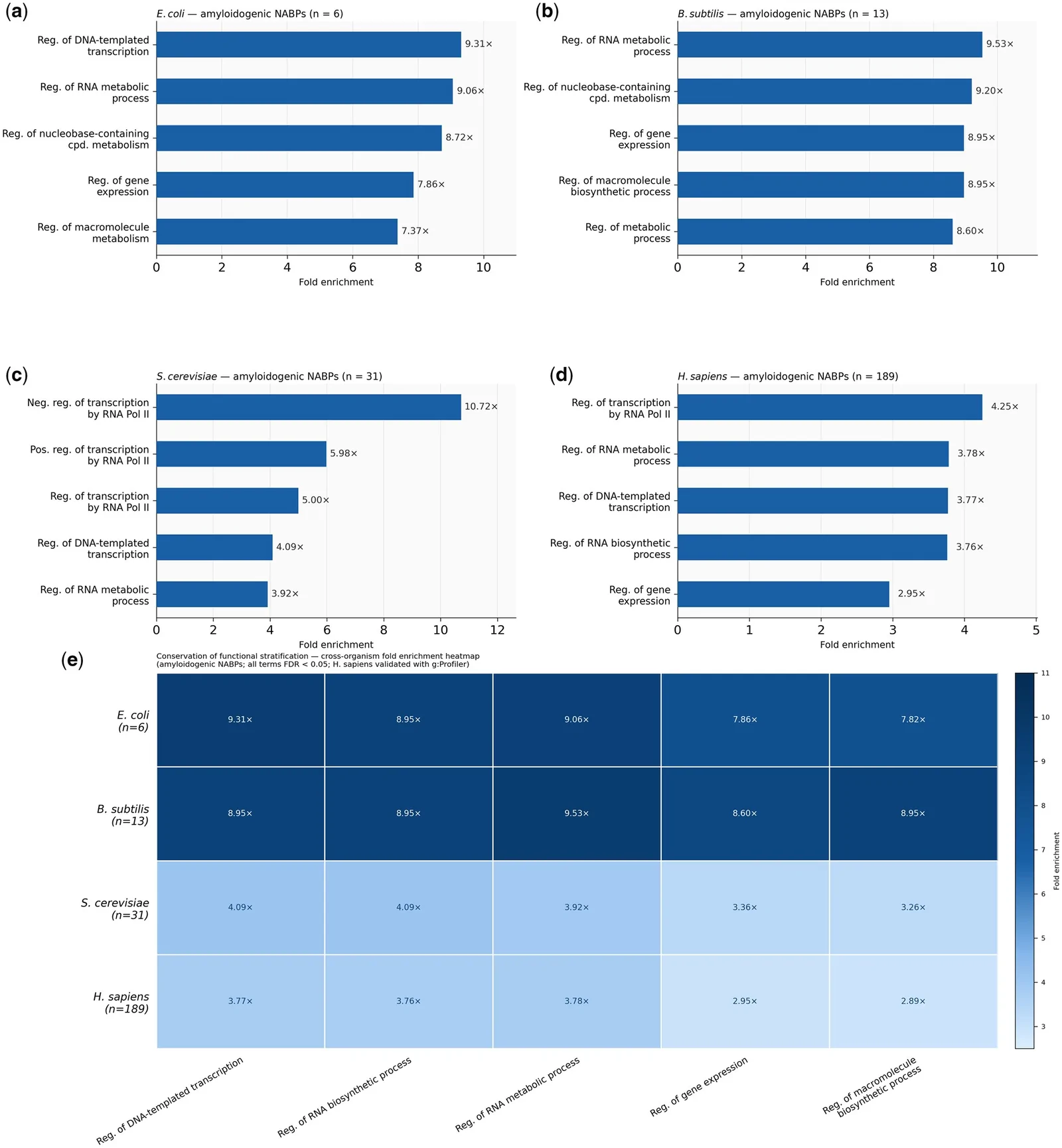
Cross-organism GO enrichment analysis of amyloidogenic nucleic-acid-binding proteins predicted by VAEBAC. (a–d) Top enriched GO biological process terms for amyloidogenic NABPs in *E. coli*, *B. subtilis*, *S. cerevisiae* and *H. sapiens* respectively. Fold enrichment values are shown for significantly enriched GO terms following FDR correction (FDR < 0.05; Fisher’s exact test, PANTHER). (e) Summary heatmap showing fold enrichment of key transcriptional regulatory GO terms across all four organisms, illustrating the conservation of functional stratification from prokaryotes to humans. GO enrichment results for *H. sapiens* were independently validated using g: Profiler and REVIGO semantic clustering (Figs 8–11, available as supplementary data at *Bioinformatics* online).

The conservation of this functional stratification pattern across organisms spanning ∼ 3.5 billion years of evolutionary divergence—from Gram-negative and Gram-positive bacteria to budding yeast and humans—indicates that selective tolerance of aggregation susceptibility within transcriptional regulatory NABPs represents a conserved organizational principle rather than an organism-specific observation. Although the number of amyloidogenic NABPs is limited in prokaryotic proteomes, the consistency of enrichment across multiple organisms and annotation frameworks supports the robustness of the observed pattern. The cross-organism fold enrichment heatmap (Fig. 5e) illustrates the consistency of transcriptional regulatory enrichment across all organisms examined.

GO enrichment results for *H. sapiens* were independently validated using g: Profiler and REVIGO semantic clustering, both confirming the dominance of transcriptional regulatory terms among amyloidogenic NABPs and the enrichment of constitutive RNA processing terms among non-amyloidogenic NABPs (Figs 8–11 and Tables 3 and 4, available as supplementary data at *Bioinformatics* online).

To provide a direct hypothesis-driven statistical test of transcription factor enrichment independent of GO annotation pipelines, Fisher’s exact test was performed for eukaryotic organisms using UniProt GO:0003700 (DNA-binding transcription factor activity) annotations. Amyloidogenic NABPs were significantly enriched among transcription regulators in both *S. cerevisiae* (odds ratio = 2.78; *P* = .005) and *H. sapiens* (odds ratio = 1.44; *P* = .010; Fig. 6), independently confirming the GO enrichment findings.

**Figure 6:**
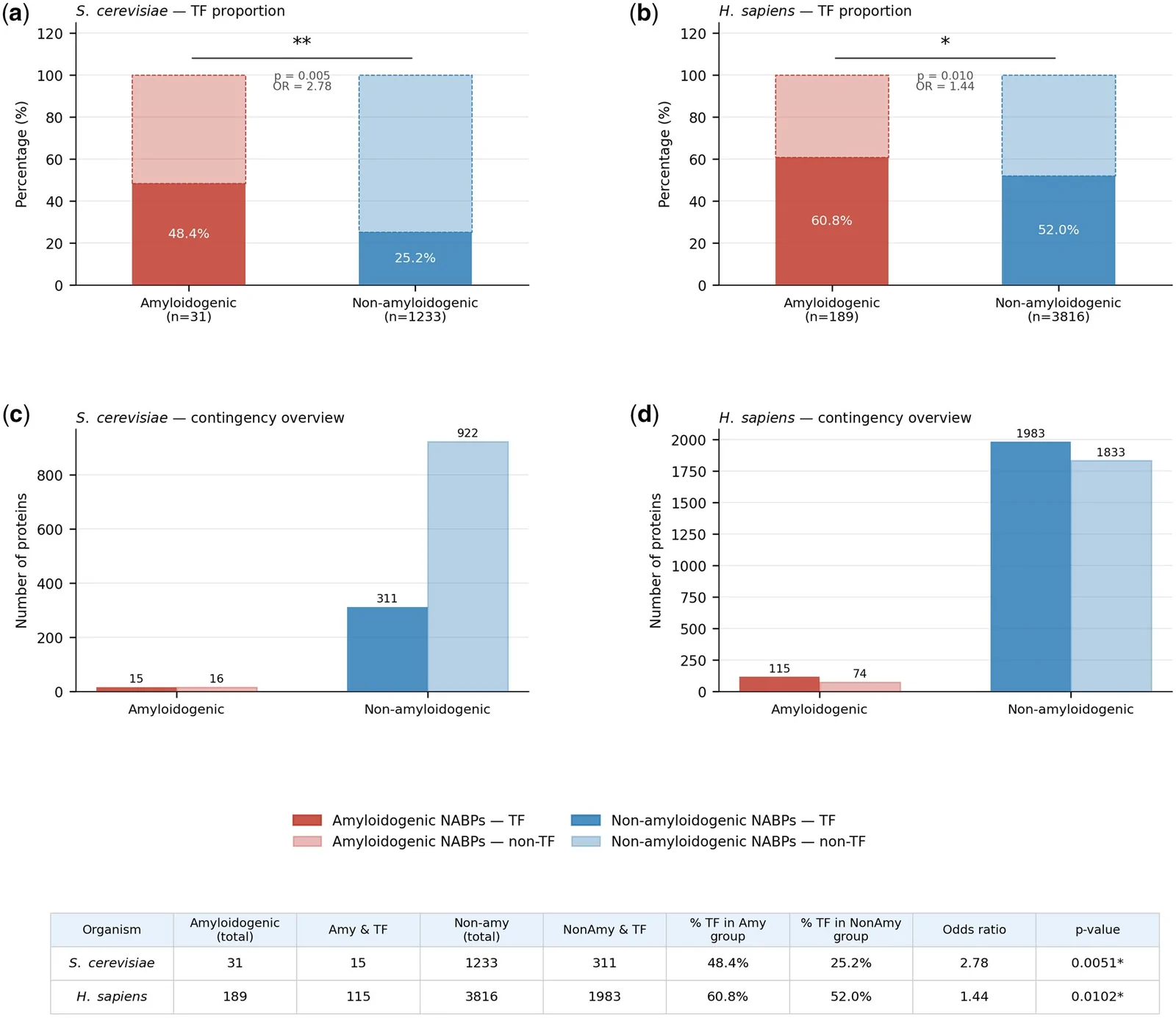
Fisher’s exact test confirms enrichment of transcription regulators among amyloidogenic NABPs in eukaryotic proteomes. (a, b) Stacked bar charts showing the proportion of transcription regulators (TF, solid fill) and non-transcription regulators among amyloidogenic and non-amyloidogenic NABPs in *S. cerevisiae* and *H. sapiens*. Significance markers and odds ratios are indicated. (c, d) Raw protein counts for each contingency group. TF classification based on GO: 0003700 (DNA-binding transcription factor activity). Fisher’s exact test, one-sided. *P<.05, **P<.01.

### 3.8 Spatial segregation of aggregation-prone regions and DNA-binding residues

Residue-level aggregation propensity and DNA-binding probability profiles were predicted using AggreProt (Planas-Iglesias *et al.* 2024) and DRNApred (Yan and Kurgan 2017), respectively, and examined for two high-confidence amyloidogenic NABPs: P0A9U6 and P17410 (ChbR) (Fig. 7). In P0A9U6, DNA-binding probability was concentrated in N-terminal and central regions (residues 22, 34, 117, and 164–168), whereas aggregation-prone regions (APRs) were restricted to specific C-terminal segments (140–142, 149–150, and 174–176), with minimal positional overlap. Similarly, for P17410 (ChbR), APRs at positions 26–29, 49–50, 72–75, 111–115, 126–130, and 149–155 were consistently found away from residues with high DNA-binding probability. Across both proteins, aggregation propensity and nucleic acid binding probability profiles exhibited complementary rather than coincident positional distributions, consistent with structural insulation of DNA-binding interfaces from aggregation-susceptible sequence segments. Residue-level predictions for individual proteins are provided in Figs 4–7, available as supplementary data at *Bioinformatics* online.

**Figure 7:**
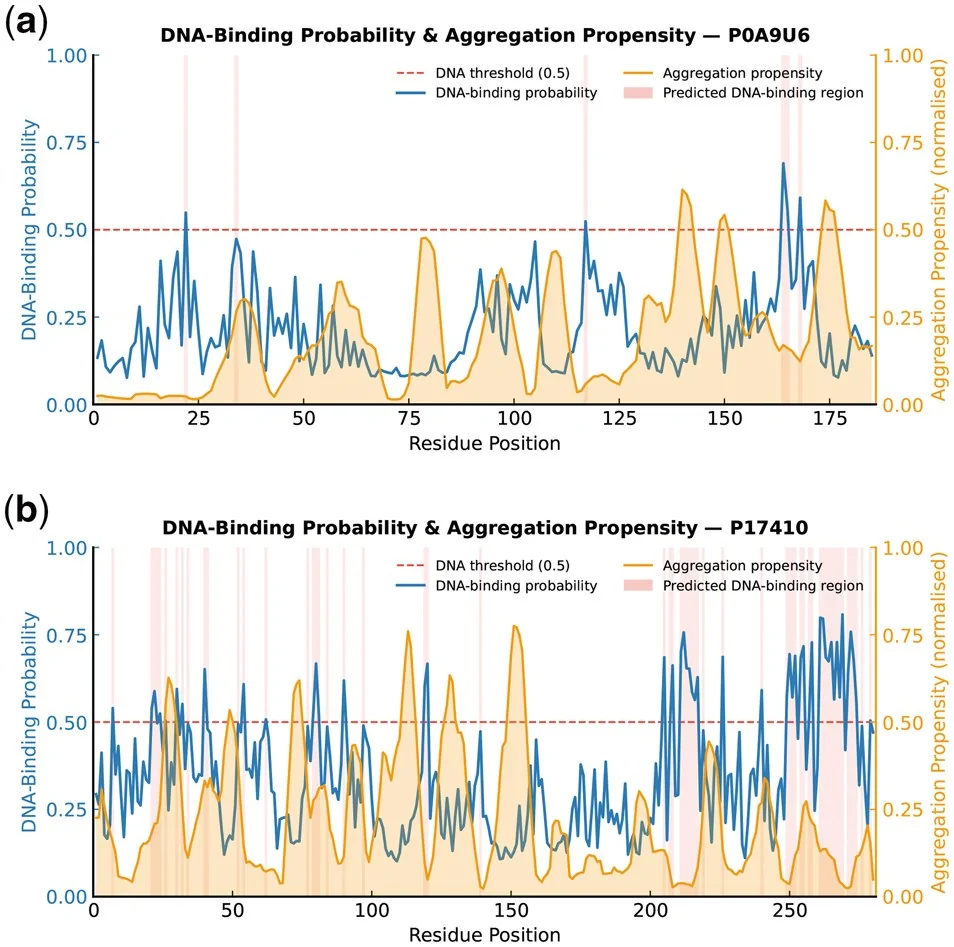
Residue-level probability profiles of aggregation propensity and DNA-binding. Comparative probability plots showing predicted aggregation-prone residues [AggreProt (Planas-Iglesias *et al.* 2024)] and DNA-binding residues [DRNApred (Yan and Kurgan, 2017)] for (a) P0A9U6 and (b) P17410 (ChbR). Aggregation peaks occur primarily outside predicted DNA-binding regions, demonstrating positional segregation of self-association propensity and nucleic-acid interaction interfaces. Per-protein residue-level profiles are provided in Figs 4–7, available as supplementary data at *Bioinformatics* online.

## 4 Discussion

This study was initially motivated by the need for robust prediction of whole-protein amyloidogenicity; however, the application of VAEBAC to the nucleic-acid-binding proteomes of four phylogenetically diverse organisms revealed a conserved organizational pattern linking aggregation propensity to transcriptional regulatory function. Specifically, NABPs predicted by VAEBAC were significantly enriched within Gene Ontology categories associated with regulation of DNA-templated transcription, RNA biosynthesis, and gene expression, despite their limited representation within the overall nucleic-acid-binding protein pool.

The functional enrichment pattern reported here was consistent across organisms spanning the full breadth of cellular life, from prokaryotes to humans. Fold enrichment values for transcriptional regulatory GO categories ranged from approximately 4–10× across organisms, with higher enrichment observed in prokaryotic proteomes where the amyloidogenic NABP sets, though smaller in absolute number, represent a more functionally coherent group. Fisher’s exact test in eukaryotic organisms independently confirmed that amyloidogenic NABPs are significantly over-represented among transcription factors annotated by GO:0003700, providing annotation-tool-independent support for the central finding.

These findings show that aggregation susceptibility may be selectively tolerated within higher-order regulatory tiers of transcriptional regulation, while remaining disfavoured within constitutive transcriptional execution machinery. Such compartmentalization follows the hypothesis that regulatory nucleic-acid-binding proteins with frequently modulated activity under stress conditions may accommodate aggregation-prone sequence elements without compromising transcriptional fidelity (Saarikangas and Barral 2016, Fassler *et al.* 2021).

Residue-level analysis of representative transcriptional regulators (P0A9U6 and P17410) further supported this inference, showing spatial separation between predicted APRs and DNA-binding residues. The inherent segregation of APR regions and DNA-binding residues reveals that sequence segments contributing to aggregation propensity may be functionally separated from nucleic-acid interaction interfaces, allowing regulatory proteins to retain binding competence under conditions promoting partial misfolding or self-association (Huang *et al.* 2013, Halfmann 2016, Afroz *et al.* 2017, Sahin *et al.* 2023).

Overall, these multi-scale observations are consistent with a model in which aggregation susceptibility is not uniformly distributed across nucleic-acid-binding proteomes but instead exhibits functional stratification aligned with transcriptional regulatory roles (De Baets *et al.* 2015, Monti *et al.* 2021). Aggregation-prone sequence features may be tolerated within modulatory regulatory proteins, where transient conformational plasticity could serve adaptive roles in stress-responsive gene expression, while being selectively minimized in proteins directly responsible for transcriptional execution.

The experimental validation of three VAEBAC-predicted amyloidogenic proteins from the *E. coli* proteome provides independent support for aggregation susceptibility in proteins involved in nucleic-acid metabolism. Among these, the small subunit of exodeoxyribonuclease VII (B1XF10), which participates in DNA repair (Lovett 2011, Repar *et al.* 2013, Huang *et al.* 2021), and pyrimidine/purine nucleoside phosphorylase (P0C037), a key enzyme in nucleoside salvage pathways (Bzowska *et al.* 2000, Pugmire and Ealick 2002, Bronckaers *et al.* 2009), both exhibited ThT fluorescence enhancement and fibrillar morphology under AFM consistent with amyloid formation.

Beyond the benchmark comparison, VAEBAC’s threshold-independent discrimination remained stable when evaluated on sequences genuinely absent from its training and benchmark data: AUC on the strictly novel Yang *et al.* subset (0.906) was comparable to AUC on the 110-sequence independent benchmark itself (0.904), despite the fixed operating threshold yielding a lower MCC on the novel subset (0.573 versus 0.734 on the benchmark). This pattern indicates that the model’s learned ranking of sequences by amyloidogenicity generalizes more consistently than its calibrated binary decision boundary; users applying VAEBAC to sequence distributions substantially different from the present training and benchmark data should therefore consider recalibrating the operating threshold rather than assuming that the benchmark-calibrated value transfers unchanged. In the separate cross-dataset evaluation against FA-Amy, VAEBAC retained 90.0% of its internal MCC compared with 58.4% for FA-Amy. We report this retention difference as secondary, descriptive evidence rather than as a primary claim of robustness, and interpret it alongside the length-correlated signal identified in our interpretability analyses (see below), which may partly contribute to retained performance across datasets. The number of VAEBAC-predicted amyloidogenic NABPs identified in each organism was limited, and functional enrichment analyses should therefore be interpreted as indicative of aggregation-associated compartmentalization rather than definitive evidence of regulatory segregation.

Although a lower, more balanced threshold can be obtained using Youden’s statistic on the independent benchmark (τ=0.43919; Table 3), proteome-scale applications require stricter control of false positives. The use of a uniform threshold of 0.95—fixed *a priori* and independently of both benchmark-derived operating points discussed above—therefore prioritizes specificity over sensitivity, ensuring that the observed enrichment patterns reflect genuine high-confidence amyloidogenic NABPs rather than artefacts of false-positive accumulation. Critically, this threshold was fixed identically across all four organisms prior to enrichment analysis, precluding post hoc adjustment to obtain desired biological signals.

Interpretability analyses of the trained classifier revealed that sequence length constitutes a substantial component of the model’s learned representation: the dominant principal component of the latent space (94.8% of variance) and the latent dimensions most influential to classifier predictions (SHAP analysis) both correlated strongly with sequence length rather than compositional features. This is not an incidental property of the fitted weights but a consequence of the architecture: because adaptive average pooling is taken over the fixed padded width rather than the true sequence length, length is written into the latent representation by construction (Methods). Consistent with this, the classifier head does not learn to discount the length-encoding dimensions—19 of its 20 most influential latent dimensions are length-correlated at |r|>0.3 (Table 12, available as supplementary data at *Bioinformatics* online). Class-wise sequence length differed substantially across three independently constructed benchmark datasets (median lengths 136–168 residues for amyloidogenic versus 564–692 residues for non-amyloidogenic sequences). A length-only classifier accounted for a meaningful portion of predictive performance (AUC 0.789–0.828 across the independent benchmark and external test sets, versus 0.904 and 0.929, respectively, for the full model); however, this difference was statistically significant on both datasets (DeLong’s test, *P* = .0055 and *P* = 1.2 × 10^–8^, respectively), confirming that VAEBAC captures substantial predictive signal beyond sequence length alone. The observed length disparity is consistent with the established tendency of amyloid- and prion-forming domains to be short, low-complexity regions, though it may also partly reflect the compositional sources of current benchmark datasets. We report this as a characteristic of the current training data; insufficient length overlap between classes precluded construction of a reliable length-matched control (Table 10, available as supplementary data at *Bioinformatics* online); the full-sample likelihood-ratio analysis reported in Results directly addresses this limitation without relying on subsetting. We additionally performed a length-stratified evaluation, which showed above-chance discrimination within every length quartile of the independent benchmark (AUC 0.731–0.917) while also showing that performance is weakest in the longest quartile (Table 13, available as supplementary data at *Bioinformatics* online). Construction of length-balanced amyloid benchmarks, in which class membership and sequence length are not confounded at source, remains the substantive remedy and a priority for future benchmark development in this field.

Several limitations of the present evaluation should be noted. First, the benchmark-calibrated threshold (τ=0.9354) reported alongside the Youden J-optimal threshold in Table 3 was obtained by scanning the independent benchmark itself and is therefore a post hoc operating point; its accuracy, sensitivity, specificity and MCC values should not be read as unbiased estimates of out-of-sample performance, and we have reported the Youden J threshold and the threshold-independent AUC alongside it for this reason. Second, of the 161 Yang *et al.* positive sequences, 27 overlap the 110-sequence benchmark and 109 overlap the training pool (136 in total), with overlap concentrated primarily in the training pool rather than the benchmark used for threshold calibration; performance on the full Yang set should therefore not be treated as a fully independent validation, though this overlap does not affect the frozen-threshold external application itself, since τ=0.9354 was selected prior to and was not re-optimized on the Yang data. The strictly novel subset we report separately is a stricter test, but contains only 25 positive sequences, limiting the precision of its MCC and sensitivity estimates. Third, threshold-dependent performance was consistently less stable across datasets than the threshold-independent AUC, indicating that a single fixed operating point may not transfer optimally to substantially different sequence distributions. Fourth, interpretability analyses indicate that sequence length is a major correlate of the model’s predictions and of benchmark composition more broadly (see above); while VAEBAC significantly outperforms a length-only baseline on both the independent benchmark and the external dataset, the extent to which reported performance reflects length-independent amyloidogenicity signal versus dataset length composition remains an open question for the field, not fully resolved by the present analysis. We identify masked mean pooling—restricting the encoder’s pooling to each sequence’s true unpadded positions—as the recommended architectural remedy for future versions of VAEBAC: applied at inference time to the present frozen model it reduces AUC by 0.036 (0.904–0.868) while improving MCC (0.660–0.687) and removing essentially all latent length encoding, though because it shifts the latent scale the classifier head was calibrated under, the resulting probabilities are uncalibrated and would require a retrained and recalibrated model to be used operationally. These limitations do not, in our view, undermine the cross-organism functional-stratification findings, the experimental validation, or the proteome-scale screening results, which do not depend on any single benchmark operating point.

The present cross-organism analysis demonstrates that functional stratification of amyloidogenic NABPs is conserved across prokaryotes and eukaryotes. Future investigations should examine whether this pattern extends to additional organisms and cellular contexts, and whether the selectively elevated aggregation propensity of regulatory NABPs is reflected in measurable differences in their conformational dynamics or stress-responsive behaviour *in vivo*. Network-level analyses across multiple organisms, and experimental characterisation of additional VAEBAC-predicted candidates, represent important directions for establishing the mechanistic basis of this conserved organisational principle.

## Supplementary Material

btag745_Supplementary_Data

## Data Availability

The source code for VAEBAC is publicly available on GitHub at https://github.com/uzairamu/VAEBAC under the MIT License. Trained model weights and associated data files are deposited on Zenodo and are freely accessible at https://doi.org/10.5281/zenodo.18872451. A web-based interface for running predictions without local installation is available at https://www.vaebac.com.

## References

[btag745-B1] Afroz T , Hock E-M, Ernst P et al Functional and dynamic polymerization of the ALS-linked protein TDP-43 antagonizes its pathologic aggregation. Nat Commun 2017;8:45. 10.1038/s41467-017-00062-0 PMC5491494 28663553

[btag745-B2] Akbey Ü , Andreasen M. Functional amyloids from bacterial biofilms—structural properties and interaction partners. Chem Sci 2022;13:6457–77. 10.1039/d2sc00645f PMC9172111 35756505

[btag745-B3] Altschul SF , Madden TL, Schäffer AA et al Gapped BLAST and PSI-BLAST: a new generation of protein database search programs. Nucleic Acids Res 1997;25:3389–402. 10.1093/nar/25.17.3389 PMC146917 9254694

[btag745-B4] Ashburner M , Ball CA, Blake JA et al Gene ontology: tool for the unification of biology. Nat Genet 2000;25:25–9. 10.1038/75556 PMC3037419 10802651

[btag745-B5] Auriemma Citarella A , Di Biasi L, De Marco F et al ENTAIL: yet another amyloid fibrils classifier. BMC Bioinformatics 2022;23:517. 10.1186/s12859-022-05070-6 PMC9714056 36456900

[btag745-B6] Bloch S , Loutfi G, Moroy G et al Bacterial amyloids as hubs for nucleic acid interactions: implications and mechanisms. Int J Mol Sci 2025;26:6560. 10.3390/ijms26146560 PMC12294510 40724810

[btag745-B7] Bronckaers A , Gago F, Balzarini J et al The dual role of thymidine phosphorylase in cancer development and chemotherapy. Med Res Rev 2009;29:903–53. 10.1002/med.20159 PMC7168469 19434693

[btag745-B8] Burdukiewicz M , Sobczyk P, Rödiger S et al Amyloidogenic motifs revealed by n-gram analysis. Sci Rep 2017;7:12961. 10.1038/s41598-017-13210-9 PMC5636826 29021608

[btag745-B9] Bzowska A , Kulikowska E, Shugar D. Purine nucleoside phosphorylases: properties, functions, and clinical aspects. Pharmacol Ther 2000;88:349–425. 10.1016/s0163-7258(00)00097-8 11337031

[btag745-B10] Caillet J , Baron B, Boni IV et al Identification of protein-protein and ribonucleoprotein complexes containing hfq. Sci Rep 2019;9:14054. 10.1038/s41598-019-50562-w PMC6773851 31575967

[btag745-B11] Candido S, Hayes T, Derry A et al Language Modeling Materializes a World Model of Protein Biology. bioRxiv. 2026.

[btag745-B12] Cech GM , Szalewska-Pałasz A, Kubiak K et al The *Escherichia coli* Hfq protein: an unattended DNA-transactions regulator. Front Mol Biosci 2016;3:36. 10.3389/fmolb.2016.00036 PMC4963395 27517037

[btag745-B13] Charoenkwan P , Ahmed S, Nantasenamat C et al AMYPred-FRL is a novel approach for accurate prediction of amyloid proteins by using feature representation learning. Sci Rep 2022;12:7697. 10.1038/s41598-022-11897-z PMC9095707 35546347

[btag745-B14] Charoenkwan P , Kanthawong S, Nantasenamat C et al iAMY-SCM: improved prediction and analysis of amyloid proteins using a scoring card method with propensity scores of dipeptides. Genomics 2021;113:689–98. 10.1016/j.ygeno.2020.09.065 33017626

[btag745-B15] Chauhan S , Ahmad S. Enabling full-length evolutionary profiles based deep convolutional neural network for predicting DNA-binding proteins from sequence. Proteins 2020;88:15–30. 10.1002/prot.25763 31228283

[btag745-B16] Chou PY , Fasman GD. Conformational parameters for amino acids in helical, β-sheet, and random coil regions calculated from proteins. Biochemistry 1974;13:211–22. 10.1021/bi00699a001 4358939

[btag745-B17] De Baets G , Van Doorn L, Rousseau F et al Increased aggregation is more frequently associated to human disease-associated mutations than to neutral polymorphisms. PLoS Comput Biol 2015;11:e1004374. 10.1371/journal.pcbi.1004374 PMC4560525 26340370

[btag745-B18] DeLong ER , DeLong DM, Clarke-Pearson DL. Comparing the areas under two or more correlated receiver operating characteristic curves: a nonparametric approach. Biometrics 1988;44:837–45. 3203132

[btag745-B19] Fassler JS , Skuodas S, Weeks DL et al Protein aggregation and disaggregation in cells and development. J Mol Biol 2021;433:167215. 10.1016/j.jmb.2021.167215 PMC8530975 34450138

[btag745-B20] Fernandez-Escamilla A-M , Rousseau F, Schymkowitz J et al Prediction of sequence-dependent and mutational effects on the aggregation of peptides and proteins. Nat Biotechnol 2004;22:1302–6. 10.1038/nbt1012 15361882

[btag745-B21] Frousios KK , Iconomidou VA, Karletidi C-M et al Amyloidogenic determinants are usually not buried. BMC Struct Biol 2009;9:44. 10.1186/1472-6807-9-44 PMC2714319 19589171

[btag745-B22] Fu L , Niu B, Zhu Z et al CD-HIT: accelerated for clustering the next-generation sequencing data. Bioinformatics 2012;28:3150–2. 10.1093/bioinformatics/bts565 PMC3516142 23060610

[btag745-B23] The Gene Ontology Consortium, Aleksander SA, Balhoff JP, Carbon S et al The gene ontology knowledgebase in 2026. Nucleic Acids Res 2026;54:D1779–92. 10.1093/nar/gkaf1292 PMC12807639 41413728

[btag745-B24] Goldschmidt L , Teng PK, Riek R et al Identifying the amylome, proteins capable of forming amyloid-like fibrils. Proc Natl Acad Sci USA 2010;107:3487–92. 10.1073/pnas.0915166107 PMC2840437 20133726

[btag745-B25] Halfmann R. A glass menagerie of low complexity sequences. Curr Opin Struct Biol 2016;38:18–25. 10.1016/j.sbi.2016.05.002 PMC5010471 27258703

[btag745-B26] Hentze MW , Castello A, Schwarzl T et al A brave new world of RNA-binding proteins. Nat Rev Mol Cell Biol 2018;19:327–41. 10.1038/nrm.2017.130 29339797

[btag745-B27] Huang S-YN , Michaels SA, Mitchell BB et al Exonuclease VII repairs quinolone-induced damage by resolving DNA gyrase cleavage complexes. Sci Adv 2021;7:eabe0384. 10.1126/sciadv.abe0384 PMC7929499 33658195

[btag745-B28] Huang Y-C , Lin K-F, He R-Y et al Inhibition of TDP-43 aggregation by nucleic acid binding. PLoS One 2013;8:e64002. 10.1371/journal.pone.0064002 PMC3667863 23737961

[btag745-B29] Hudson WH , Ortlund EA. The structure, function and evolution of proteins that bind DNA and RNA. Nat Rev Mol Cell Biol 2014;15:749–60. 10.1038/nrm3884 PMC4280011 25269475

[btag745-B30] Karas VO , Westerlaken I, Meyer AS. The DNA-binding protein from starved cells (Dps) utilizes dual functions to defend cells against multiple stresses. J Bacteriol 2015;197:3206–15. 10.1128/JB.00475-15 PMC4560292 26216848

[btag745-B31] Knowles TP , Vendruscolo M, Dobson CM. The amyloid state and its association with protein misfolding diseases. Nat Rev Mol Cell Biol 2014;15:384–96. 10.1038/nrm3810 24854788

[btag745-B32] Kolberg L , Raudvere U, Kuzmin I et al g: profiler—interoperable web service for functional enrichment analysis and gene identifier mapping (2023 update). Nucleic Acids Res 2023; 51:W207–W212. 10.1093/nar/gkad347 PMC10320099 37144459

[btag745-B33] Kosolapova AO , Antonets KS, Belousov MV et al Biological functions of prokaryotic amyloids in interspecies interactions: facts and assumptions. Int J Mol Sci 2020;21:7240. 10.3390/ijms21197240 PMC7582709 33008049

[btag745-B34] Krishnan R , Lindquist SL. Structural insights into a yeast prion illuminate nucleation and strain diversity. Nature 2005;435:765–72. 10.1038/nature03679 PMC1405905 15944694

[btag745-B35] Kyte J , Doolittle RF. A simple method for displaying the hydropathic character of a protein. J Mol Biol 1982;157:105–32. 10.1016/0022-2836(82)90515-0 7108955

[btag745-B36] Li Y , Zhang Z, Teng Z et al PredAmyl-MLP: prediction of amyloid proteins using multilayer perceptron. Comput Math Methods Med 2020;2020:8845133. 10.1155/2020/8845133 PMC7700051 33294004

[btag745-B37] Louros N , Orlando G, De Vleeschouwer M et al Structure-based machine-guided mapping of amyloid sequence space reveals uncharted sequence clusters with higher solubilities. Nat Commun 2020;11:3314. 10.1038/s41467-020-17207-3 PMC7335209 32620861

[btag745-B38] Lovett ST. The DNA exonucleases of *Escherichia coli*. EcoSal Plus 2011;4. 10.1128/ecosalplus.4.4.7 PMC4238392 26442508

[btag745-B39] Lundberg SM , Lee SI. A unified approach to interpreting model predictions. Adv Neural Inf Process Syst 2017;30:4765–74.

[btag745-B40] Martinez A , Kolter R. Protection of DNA during oxidative stress by the nonspecific DNA-binding protein dps. J Bacteriol 1997;179:5188–94. 10.1128/jb.179.16.5188-5194.1997 PMC179379 9260963

[btag745-B41] Monti M , Armaos A, Fantini M et al Aggregation is a context-dependent constraint on protein evolution. Front Mol Biosci 2021;8:678115. 10.3389/fmolb.2021.678115 PMC8249573 34222334

[btag745-B42] Navarro S , Ventura S. Computational methods to predict protein aggregation. Curr Opin Struct Biol 2022;73:102343. 10.1016/j.sbi.2022.102343 35240456

[btag745-B43] Niu M , Li Y, Wang C et al RFAmyloid: a web server for predicting amyloid proteins. Int J Mol Sci 2018;19:2071. 10.3390/ijms19072071 PMC6073578 30013015

[btag745-B44] Otzen D , Riek R. Functional amyloids. Cold Spring Harb Perspect Biol 2019;11:a033860. 10.1101/cshperspect.a033860 PMC6886451 31088827

[btag745-B45] Pawlicki S , Le Béchec A, Delamarche C. AMYPdb: a database dedicated to amyloid precursor proteins. BMC Bioinformatics 2008;9:273. 10.1186/1471-2105-9-273 PMC2442844 18544157

[btag745-B46] Pedregosa F et al Scikit-learn: machine learning in python. J Mach Learn Res 2011;12:2825–30.

[btag745-B47] Planas-Iglesias J , Borko S, Swiatkowski J et al AggreProt: a web server for predicting and engineering aggregation prone regions in proteins. Nucleic Acids Res 2024;52:W159–69. 10.1093/nar/gkae420 PMC11223854 38801076

[btag745-B48] Pugmire MJ , Ealick SE. Structural analyses reveal two distinct families of nucleoside phosphorylases. Biochem J 2002;361:1–25. 10.1042/0264-6021:3610001 PMC1222293 11743878

[btag745-B49] Redler RL , Shirvanyants D, Dagliyan O et al Computational approaches to understanding protein aggregation in neurodegeneration. J Mol Cell Biol 2014;6:104–15. 10.1093/jmcb/mju007 PMC3995224 24620031

[btag745-B50] Repar J , Briški N, Buljubašić M et al Exonuclease VII is involved in “reckless” DNA degradation in UV-irradiated *Escherichia coli*. Mutat Res 2013;750:96–104. 10.1016/j.mrgentox.2012.10.005 23123979

[btag745-B51] Saarikangas J , Barral Y. Protein aggregation as a mechanism of adaptive cellular responses. Curr Genet 2016;62:711–24. 10.1007/s00294-016-0596-0 27032776

[btag745-B52] Sahin C , Motso A, Gu X et al Mass spectrometry of RNA-binding proteins during liquid–liquid phase separation reveals distinct assembly mechanisms and droplet architectures. J Am Chem Soc 2023;145:10659–68. 10.1021/jacs.3c00932 PMC10197120 37145883

[btag745-B53] Supek F , Bošnjak M, Škunca N et al REVIGO summarizes and visualizes long lists of gene ontology terms. PLoS One 2011;6:e21800. 10.1371/journal.pone.0021800 PMC3138752 21789182

[btag745-B54] Thomas PD , Ebert D, Muruganujan A et al PANTHER: making genome-scale phylogenetics accessible to all. Protein Sci 2022;31:8–22. 10.1002/pro.4218 PMC8740835 34717010

[btag745-B55] Thompson M , Martín M, Olmo TS et al Massive experimental quantification allows interpretable deep learning of protein aggregation. Sci Adv 2025;11:eadt5111. 10.1126/sciadv.adt5111 PMC12042874 40305601

[btag745-B56] Tien MZ , Meyer AG, Sydykova DK et al Maximum allowed solvent accessibilities of residues in proteins. PLoS One 2013;8:e80635. 10.1371/journal.pone.0080635 PMC3836772 24278298

[btag745-B57] The UniProt Consortium. UniProt: the universal protein knowledgebase in 2023. Nucleic Acids Res 2023; 51:D523–31. 10.1093/nar/gkac1052 PMC9825514 36408920

[btag745-B58] Uversky VN , Gillespie JR, Fink AL. Why are “natively unfolded” proteins unstructured under physiologic conditions? Proteins 2000;41:415–27. 10.1002/1097-0134(20001115)41:3<415::aid-prot130>3.0.co;2-7 11025552

[btag745-B59] Van Gerven N , Van der Verren SE, Reiter DM et al The role of functional amyloids in bacterial virulence. J Mol Biol 2018;430:3657–84. 10.1016/j.jmb.2018.07.010 PMC6173799 30009771

[btag745-B60] Varadi M , De Baets G, Vranken WF et al AmyPro: a database of proteins with validated amyloidogenic regions. Nucleic Acids Res 2018; 46:D387–92. 10.1093/nar/gkx950 PMC5753394 29040693

[btag745-B61] Virtanen P , Gommers R, Oliphant TE, et al; SciPy 1.0 Contributors. SciPy 1.0: fundamental algorithms for scientific computing in python. Nat Methods 2020;17:261–72. 10.1038/s41592-019-0686-2 PMC7056644 32015543

[btag745-B62] Vogel J , Luisi BF. Hfq and its constellation of RNA. Nat Rev Microbiol 2011;9:578–89. 10.1038/nrmicro2615 PMC4615618 21760622

[btag745-B63] Wang J , Geng A, Yan Z et al FA-Amy: an amyloid protein prediction model based on protein pre-trained large models and an attention-fusion strategy. Int J Biol Macromol 2026;335:149267. 10.1016/j.ijbiomac.2025.149267 41319778

[btag745-B64] Wilson CJ , Bommarius AS, Champion JA et al Biomolecular assemblies: moving from observation to predictive design. Chem Rev 2018;118:11519–74. 10.1021/acs.chemrev.8b00038 PMC6650774 30281290

[btag745-B65] Wright PE , Dyson HJ. Intrinsically disordered proteins in cellular signalling and regulation. Nat Rev Mol Cell Biol 2015;16:18–29. 10.1038/nrm3920 PMC4405151 25531225

[btag745-B66] Yan J , Kurgan L. DRNApred, fast sequence-based method that accurately predicts and discriminates DNA- and RNA-binding residues. Nucleic Acids Res 2017;45:e84. 10.1093/nar/gkx059 PMC5449545 28132027

[btag745-B67] Yang R , Liu J, Zhang L. ECAmyloid: an amyloid predictor based on ensemble learning and comprehensive sequence-derived features. Comput Biol Chem 2023;104:107853. 10.1016/j.compbiolchem.2023.107853 36990028

