# Supplementary material for: VAEBAC: a representation-learning framework for proteome-scale prediction of amyloidogenic proteins and functional stratification of nucleic-acid-binding proteins
Source: Bioinformatics. 2026 Oct 7;42(10):btag745. doi: 10.1093/bioinformatics/btag745 (PMC13682611; doi:10.1093/bioinformatics/btag745)
Supplement: Supplementary file 1 [file btag745_supplementary_data.pdf]

Supplementary Information

VAEBAC: A representation-learning framework for proteome-scale prediction of amyloidogenic proteins and functional stratification of nucleic-acid-binding proteins

Muhammad Uzair Ashraf, Owais Ahmad, Rizwan Hasan Khan

Supplementary Methods

VAEBAC Architecture Details

Detailed specifications of the encoder, decoder, and classifier sub-networks of the VAEBAC framework are provided in Tables S1 and S2.

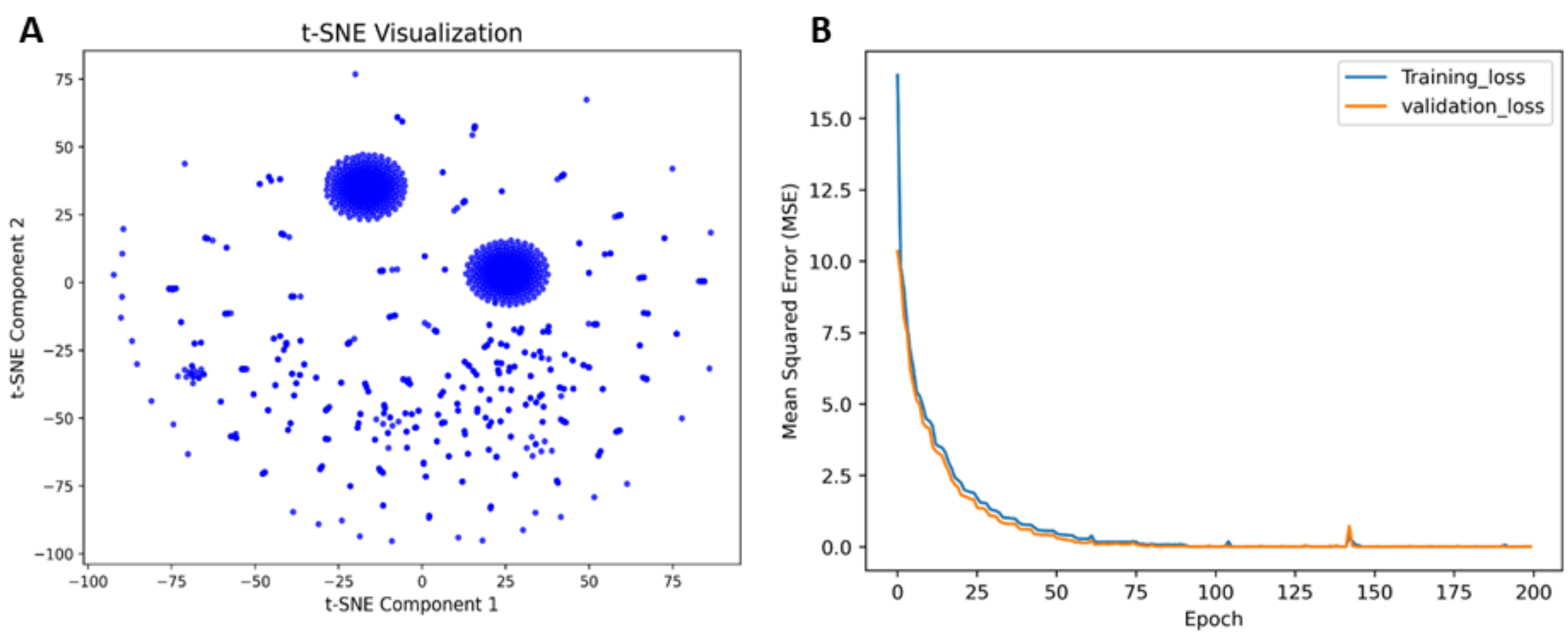

Figure S1: Latent space characterisation and training dynamics. **(A)** t-SNE visualisation of latent representations learned by the variational autoencoder. **(B)** Training and validation loss curves for variational autoencoder pre-training.

Table S1: Architecture of the VAEBAC encoder.

Component	Description
Input representation 1	One-hot encoded amino acid sequence (23 features per residue)
Input representation 2	Physicochemical feature embedding (4 features per residue)
Sequence modelling layer	LSTM (hidden size 16, 1 layer)
Physicochemical modelling layer	LSTM (hidden size 16, 1 layer)
Feature projection	Conv1D ($16 \rightarrow 12$, kernel size 1)
Feature fusion	Channel-wise concatenation
Pooling	Adaptive average pooling
Fully connected layer	Linear ($24 \rightarrow 28$)
Latent mean (μ)	Linear ($28 \rightarrow 512 / 1024$)
Latent variance ($\log \sigma^2$)	Linear ($28 \rightarrow 512 / 1024$)

Table S2: Decoder and classifier architecture.

Model stage	Architecture
Decoder (VAE pre-training)	FC (latent $\rightarrow 28 \rightarrow 24$) + Conv1D + LSTM + sigmoid
Reconstruction layers	Conv1D ($24 \rightarrow 12$) $\rightarrow$ Conv1D ($12 \rightarrow 16$)
Sequence reconstruction	LSTM (hidden size 23) + Conv1D
Classifier head	MLP ($512 \rightarrow 256 \rightarrow 256 \rightarrow 1$)
Activation functions	LeakyReLU
Output	Probability of amyloidogenicity

Supplementary Figures — Experimental Validation

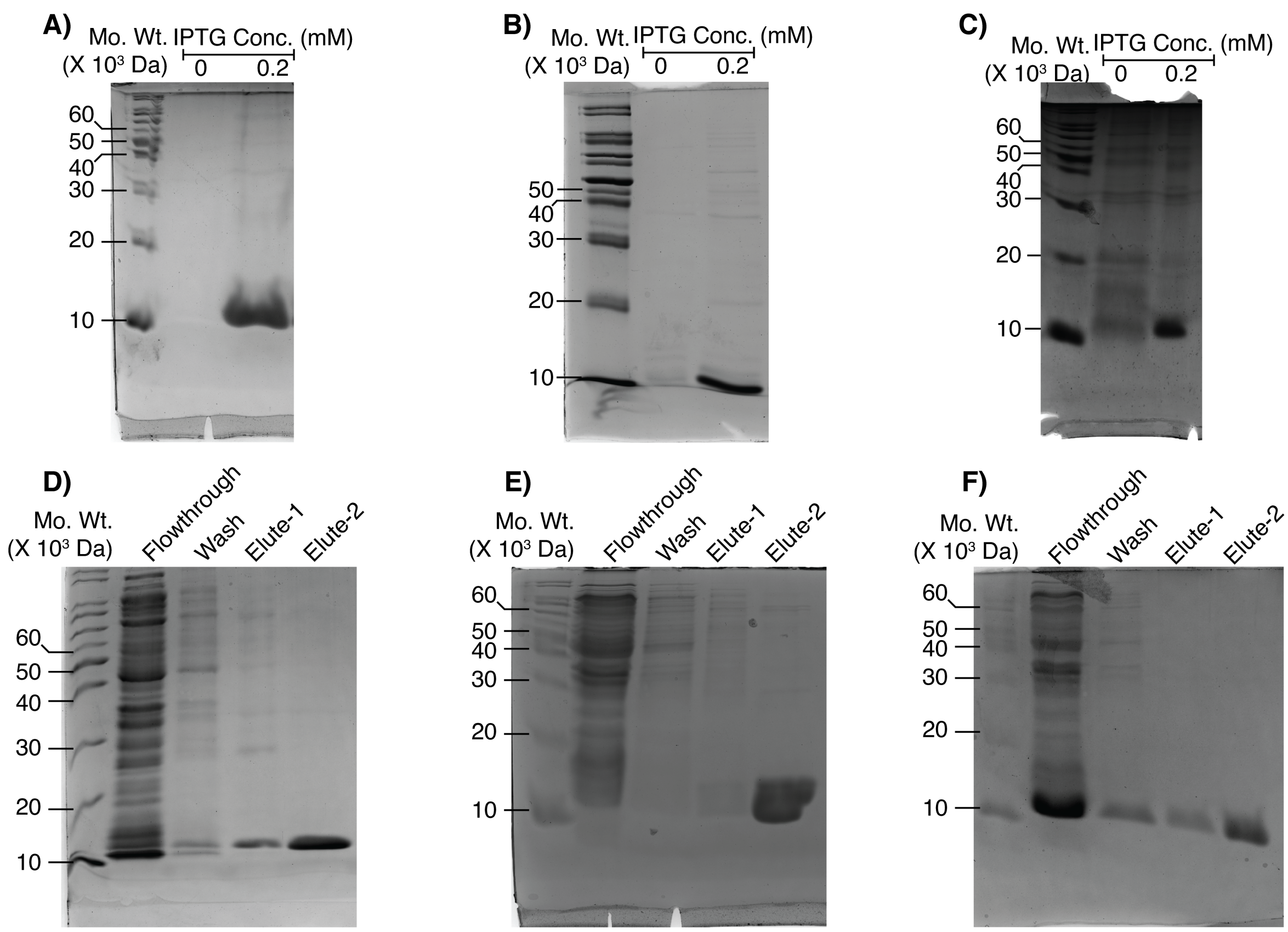

Figure S2: Expression and purification of recombinant proteins. (A–C) SDS–PAGE analysis of recombinant protein expression in *E. coli* BL21 cells in the absence (0 mM) and presence (0.2 mM) of IPTG induction; (A) B1XF10, (B) P0AA04, (C) P0C037. **(D–F)** SDS–PAGE analysis of Ni-NTA affinity purification fractions for the expressed proteins. Lanes correspond to flowthrough, wash, and elution fractions as indicated above each gel; (D) B1XF10, (E) P0AA04, (F) P0C037.

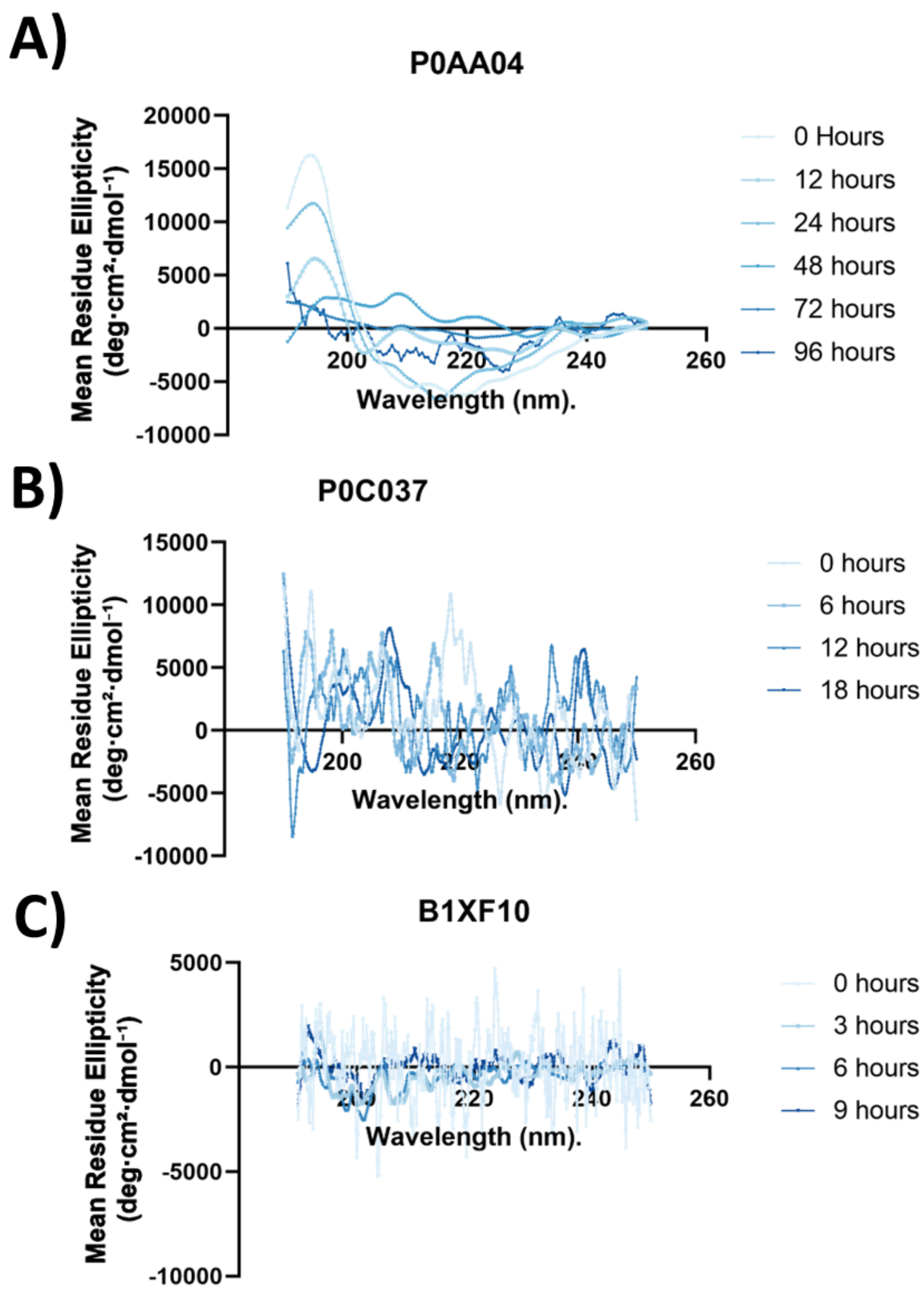

Figure S3: Time-dependent circular dichroism spectra during protein aggregation. Far-UV circular dichroism (CD) spectra recorded at different incubation time points during aggregation assays for the three proteins. **(A)** P0AA04, **(B)** P0C037, **(C)** B1XF10.

Supplementary Figures — Residue-Level Predictions

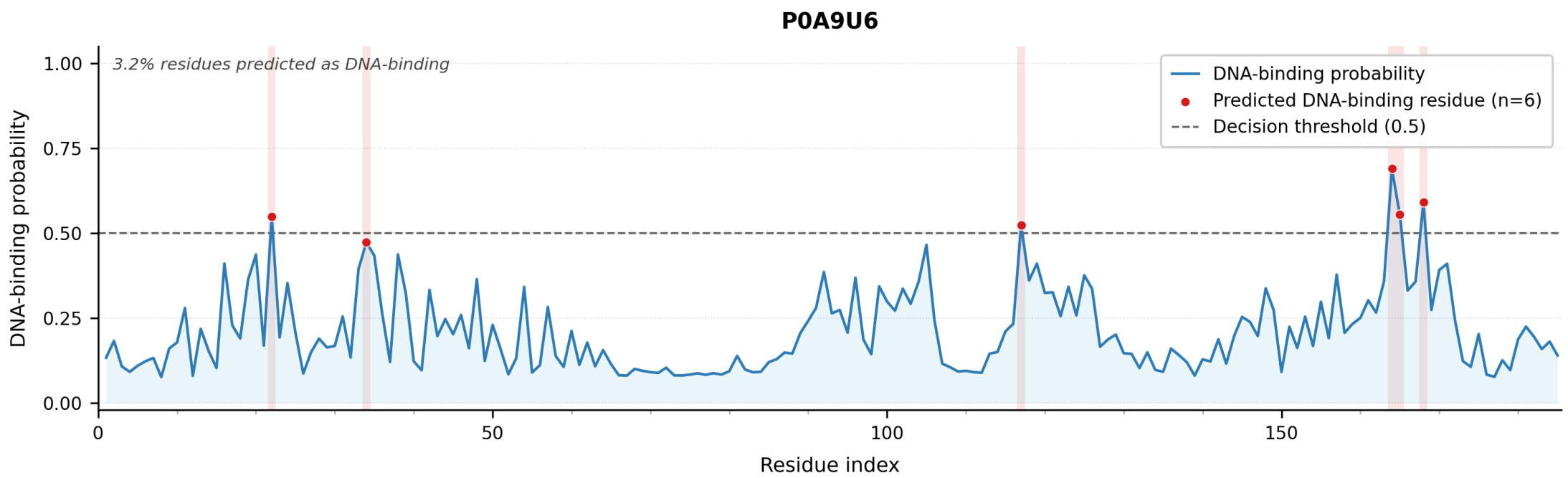

Figure S4: Per-residue DNA-binding probability profile for P0A9U6 predicted using the DRNApred server (threshold = 0.5). Residues predicted to participate in DNA binding are indicated by binary classification above the probability threshold.

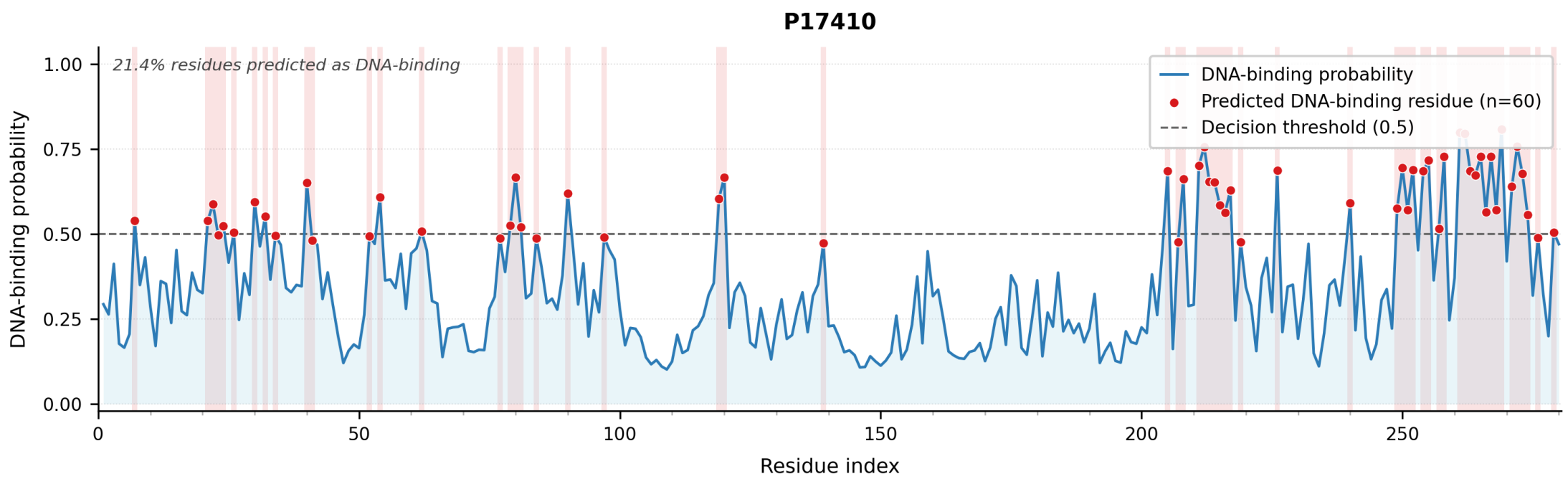

Figure S5: Per-residue DNA-binding probability profile for P17410 predicted using the DRNApred server (threshold = 0.5). Residues predicted to participate in DNA binding are indicated by binary classification above the probability threshold.

Aggregation Propensity Profile

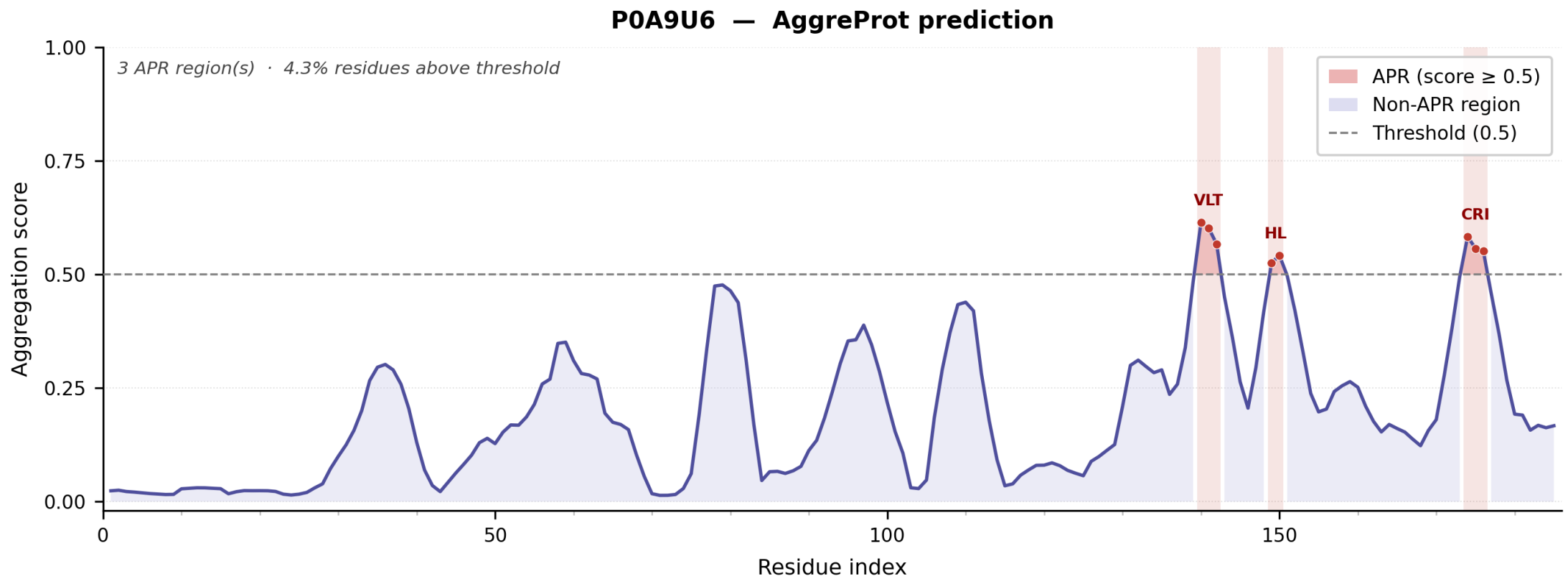

APR Regions — P0A9U6

APR #	Start	End	Length (aa)	Sequence	Max Score	Mean Score
1	140	142	3	VLT	0.6145	0.5944
2	149	150	2	HL	0.5422	0.5337
3	174	176	3	CRI	0.5835	0.5643

Figure S6: Residue-level aggregation propensity profile for P0A9U6 predicted using the AggreProt server. Aggregation scores are plotted as a function of residue index, with the dashed horizontal line indicating the threshold of 0.5 used to define aggregation-prone regions (APRs). Residues exceeding the threshold are highlighted.

Aggregation Propensity Profile

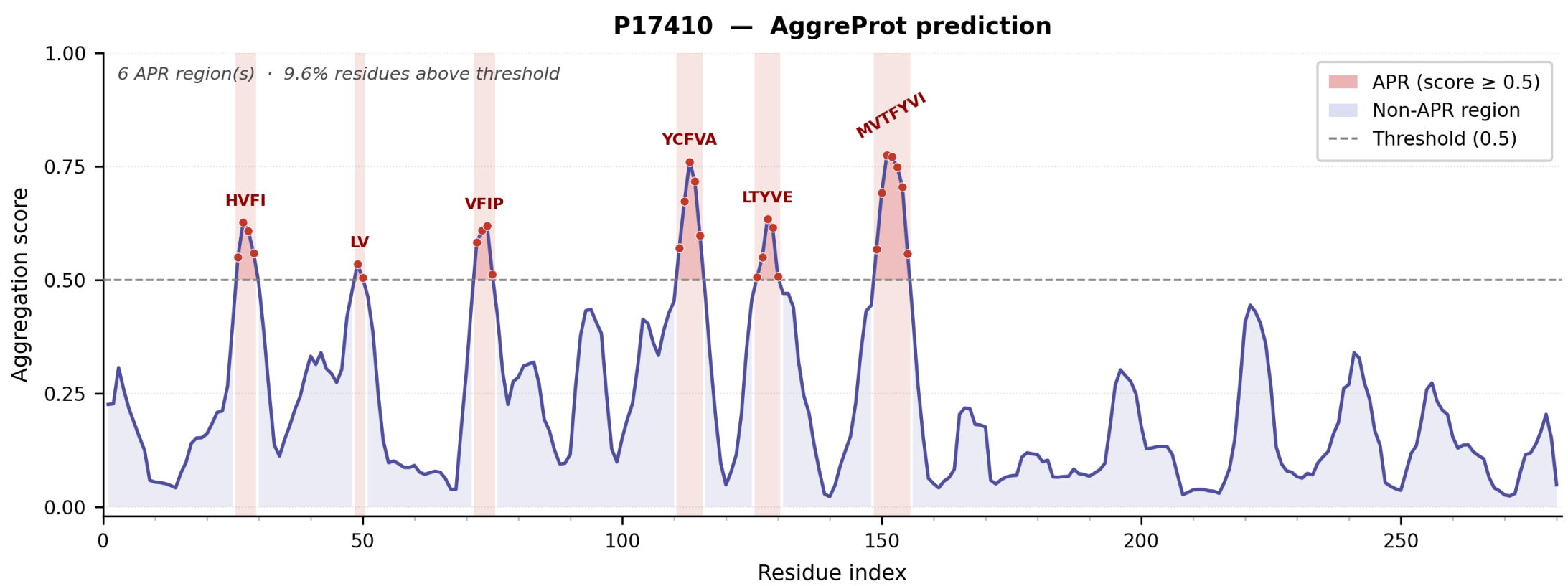

APR Regions — P17410

APR #	Start	End	Length (aa)	Sequence	Max Score	Mean Score
1	26	29	4	HVFI	0.6265	0.5859
2	49	50	2	LV	0.5343	0.5196
3	72	75	4	VFIP	0.6187	0.5807
4	111	115	5	YCFVA	0.7601	0.6634
5	126	130	5	LTYVE	0.6338	0.5621
6	149	155	7	MVTFYVI	0.7751	0.6879

Figure S7: Residue-level aggregation propensity profile for P17410 predicted using the AggreProt server. Aggregation scores are plotted as a function of residue index, with the dashed horizontal line indicating the threshold of 0.5 used to define aggregation-prone regions (APRs). Residues exceeding the threshold are highlighted.

Supplementary Figures — Cross-Tool GO Enrichment Validation (*H. sapiens*)

To provide rigorous, tool-independent validation of the GO enrichment patterns identified by PANTHER, we performed two additional, orthogonal analyses on the human amyloidogenic (n = 189) and non-amyloidogenic (n = 3816) NABP sets.

g:Profiler analysis. Enrichment was computed independently using g:Profiler (version `e113_eg59_p19_6be52918`; run 18 March 2026), which employs a distinct statistical framework (g:SCS multiple-testing correction) and a separately maintained annotation database.

REVIGO semantic clustering. Because standard GO enrichment outputs contain many closely related, redundant terms that can inflate the apparent size of a signal, we further processed the g:Profiler results through REVIGO (*REduce & VIsualize Gene Ontology*). REVIGO applies semantic similarity measures to collapse hierarchically overlapping GO terms into a compact, non-redundant representative set, thereby providing the most stringent possible summary of functional enrichment. Critically, this step removes the artefact of a single biological theme appearing inflated simply because many ontologically similar terms all pass the significance threshold.

Convergent conclusion. All three tools — PANTHER, g:Profiler, and REVIGO — independently converge on the same result: *regulation of transcription by RNA polymerase II* and related transcriptional regulatory functions constitute the dominant, non-redundant biological theme of amyloidogenic NABPs in *H. sapiens*. The persistence of this signal after REVIGO's aggressive redundancy removal confirms that the enrichment reflects a genuine, coherent functional category rather than a statistical artefact of GO hierarchy inflation. This three-way cross-tool concordance provides strong, annotation-tool-independent support for the cross-organism conservation pattern reported in the main text.

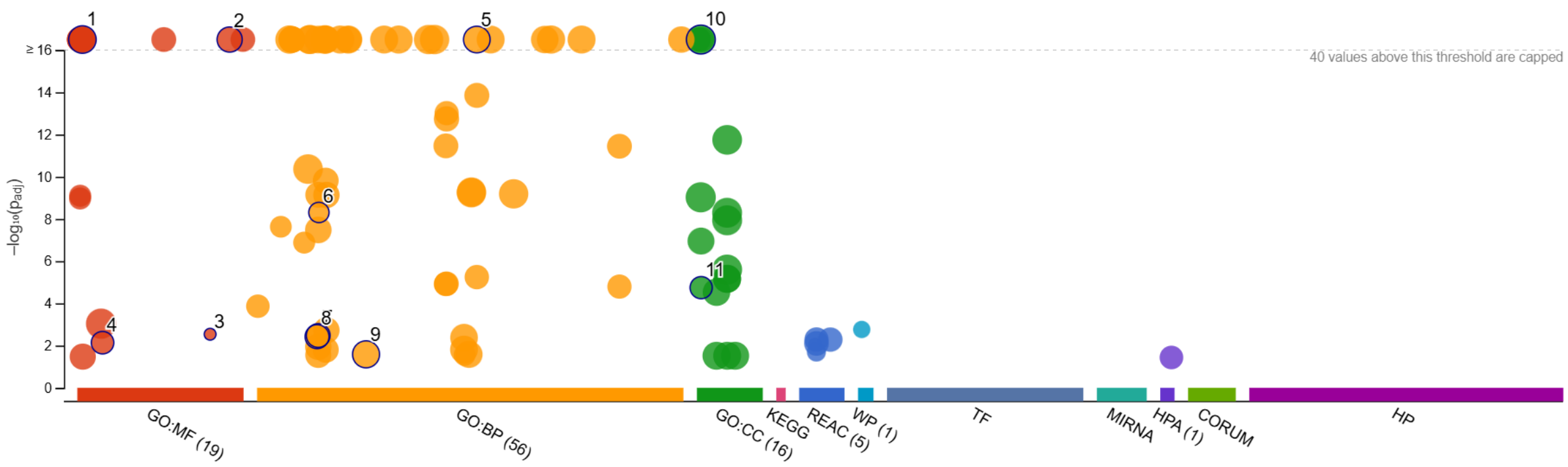

ID	Source	Term ID	Term Name	p_{adj} (query_1)
1	GO:MF	GO:0003676	nucleic acid binding	2.856×10^{-79}
2	GO:MF	GO:0140110	transcription regulator activity	3.181×10^{-44}
3	GO:MF	GO:0070974	POU domain binding	2.899×10^{-3}
4	GO:MF	GO:0008134	transcription factor binding	7.281×10^{-3}
5	GO:BP	GO:0051252	regulation of RNA metabolic process	2.748×10^{-47}
6	GO:BP	GO:0009952	anterior/posterior pattern specification	4.926×10^{-9}
7	GO:BP	GO:0009887	animal organ morphogenesis	3.327×10^{-3}
8	GO:BP	GO:0009790	embryo development	3.778×10^{-3}
9	GO:BP	GO:0030154	cell differentiation	2.596×10^{-2}
10	GO:CC	GO:0005634	nucleus	2.018×10^{-30}
11	GO:CC	GO:0005667	transcription regulator complex	1.802×10^{-5}

**version**	e113_eg59_p19_6be52918
**date**	3/18/2026, 10:25:15 AM
**organism**	hsapiens

g:Profiler

Figure S8: g:Profiler Manhattan plot for amyloidogenic human NABPs (n = 189). Each bubble represents a significantly enriched term ($p_{\text{adj}} < 0.05$, g:SCS correction) coloured by source database (GO:MF = dark orange, GO:BP = light orange, GO:CC = teal/green, KEGG = cyan, REAC = blue, TF = dark blue, MIRNA = lilac, HPA = magenta, CORUM = dark green, HP = purple). Bubble size is proportional to term size. Numbered bubbles correspond to the top-ranked terms listed in Table S3. Analysis run: g:Profiler version e113_eg59_p19_6be52918, 18 March 2026, organism: *Homo sapiens*.

Table S3: Top g:Profiler enrichment terms for amyloidogenic human NABPs ($n = 189$). Shown are the ten most significant GO:MF and GO:BP terms. Intersection/query: number of query proteins annotated to the term out of the total query size used by g:Profiler after background filtering. p_{adj}: FDR-corrected p-value (g:SCS algorithm).

Rank	Source	Term name	Term ID	$p_{\mathbf{adj}}$
Molecular Function (GO:MF)				
1	GO:MF	nucleic acid binding	GO:0003676	2.86×10^{-79}
2	GO:MF	DNA binding	GO:0003677	1.80×10^{-63}
3	GO:MF	transcription regulator activity	GO:0140110	3.18×10^{-44}
4	GO:MF	sequence-specific DNA binding	GO:0043565	4.27×10^{-43}
5	GO:MF	sequence-specific double-stranded DNA binding	GO:1990837	4.69×10^{-43}
6	GO:MF	DNA-binding transcription factor activity	GO:0003700	7.26×10^{-43}
7	GO:MF	double-stranded DNA binding	GO:0003690	3.09×10^{-42}
8	GO:MF	transcription cis-regulatory region binding	GO:0000976	5.73×10^{-41}
9	GO:MF	transcription regulatory region nucleic acid binding	GO:0001067	6.01×10^{-41}
10	GO:MF	RNA Pol II transcription regulatory region sequence-specific DNA binding	GO:0000977	1.14×10^{-39}
Biological Process (GO:BP)				
1	GO:BP	regulation of RNA metabolic process	GO:0051252	2.75×10^{-47}
2	GO:BP	regulation of nucleobase-containing compound metabolic process	GO:0019219	5.46×10^{-45}
3	GO:BP	regulation of primary metabolic process	GO:0080090	2.00×10^{-41}
4	GO:BP	DNA-templated transcription	GO:0006351	3.94×10^{-41}
5	GO:BP	regulation of DNA-templated transcription	GO:0006355	1.35×10^{-40}
6	GO:BP	regulation of RNA biosynthetic process	GO:2001141	2.22×10^{-40}
7	GO:BP	regulation of transcription by RNA polymerase II	GO:0006357	2.82×10^{-39}
8	GO:BP	transcription by RNA polymerase II	GO:0006366	1.42×10^{-38}
9	GO:BP	regulation of gene expression	GO:0010468	6.56×10^{-35}
10	GO:BP	regulation of macromolecule biosynthetic process	GO:0010556	4.62×10^{-34}

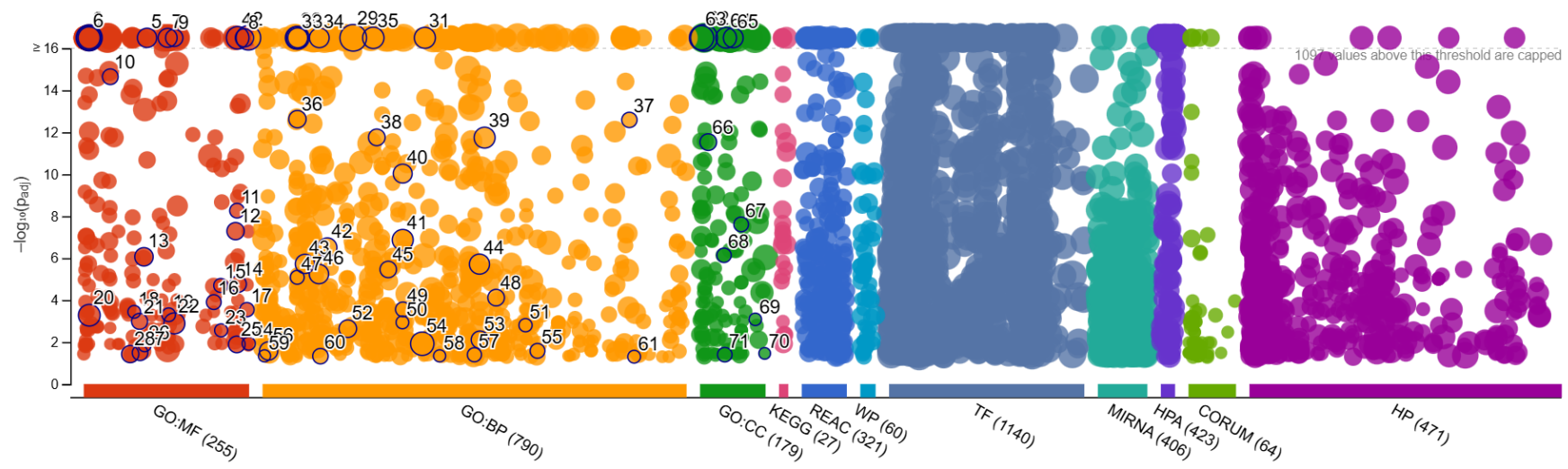

ID	Source	Term ID	Term Name	p_{adj} (query_1)
1	GO:MF	GO:0003723	RNA binding	4.941×10^{-324}
2	GO:MF	GO:1990837	sequence-specific double-stranded DNA binding	4.941×10^{-324}
3	GO:MF	GO:0003700	DNA-binding transcription factor activity	4.941×10^{-324}
4	GO:MF	GO:0140640	catalytic activity, acting on a nucleic acid	3.117×10^{-195}
5	GO:MF	GO:0031490	chromatin DNA binding	4.842×10^{-46}
6	GO:MF	GO:0003712	transcription coregulator activity	1.883×10^{-25}
7	GO:MF	GO:0042826	histone deacetylase binding	2.227×10^{-20}
8	GO:MF	GO:0180051	translation factor activity	1.792×10^{-19}
9	GO:MF	GO:0045182	translation regulator activity	4.614×10^{-19}
10	GO:MF	GO:0008327	methyl-CpG binding	2.267×10^{-15}
11	GO:MF	GO:0140517	protein-RNA adaptor activity	5.477×10^{-9}
12	GO:MF	GO:0140223	general transcription initiation factor activity	5.158×10^{-8}
13	GO:MF	GO:0030527	structural constituent of chromatin	8.641×10^{-7}
14	GO:MF	GO:0140693	molecular condensate scaffold activity	1.722×10^{-5}
15	GO:MF	GO:0097157	pre-mRNA intronic binding	2.009×10^{-5}
16	GO:MF	GO:0070182	DNA polymerase binding	1.247×10^{-4}
17	GO:MF	GO:1990226	histone methyltransferase binding	2.852×10^{-4}
18	GO:MF	GO:0017151	DEAD/H-box RNA helicase binding	3.624×10^{-4}
19	GO:MF	GO:0043047	single-stranded telomeric DNA binding	5.002×10^{-4}
20	GO:MF	GO:0003735	structural constituent of ribosome	5.249×10^{-4}
21	GO:MF	GO:0019239	deaminase activity	1.030×10^{-3}
22	GO:MF	GO:0045296	cadherin binding	1.291×10^{-3}
23	GO:MF	GO:0097322	7SK snRNA binding	2.765×10^{-3}
24	GO:MF	GO:1990948	ubiquitin ligase inhibitor activity	1.172×10^{-2}
25	GO:MF	GO:0140463	chromatin-protein adaptor activity	1.298×10^{-2}
26	GO:MF	GO:0030619	U1 snRNA binding	1.439×10^{-2}
27	GO:MF	GO:0019789	SUMO transferase activity	3.252×10^{-2}
28	GO:MF	GO:0016706	2-oxoglutarate-dependent dioxygenase activity	3.742×10^{-2}
29	GO:BP	GO:0019219	regulation of nucleobase-containing compound m...	4.941×10^{-324}
30	GO:BP	GO:0006412	translation	8.228×10^{-131}
31	GO:BP	GO:0042254	ribosome biogenesis	4.524×10^{-113}
32	GO:BP	GO:0006281	DNA repair	4.085×10^{-77}
33	GO:BP	GO:0006403	RNA localization	5.781×10^{-48}
34	GO:BP	GO:0009451	RNA modification	8.003×10^{-41}
35	GO:BP	GO:0030522	intracellular receptor signaling pathway	1.454×10^{-22}
36	GO:BP	GO:0006383	transcription by RNA polymerase III	2.368×10^{-13}
37	GO:BP	GO:1903241	U2-type prespliceosome assembly	2.580×10^{-13}
38	GO:BP	GO:0031124	mRNA 3'-end processing	1.752×10^{-12}
39	GO:BP	GO:0051607	defense response to virus	1.808×10^{-12}
40	GO:BP	GO:0034502	protein localization to chromosome	9.387×10^{-11}
41	GO:BP	GO:0034504	protein localization to nucleus	1.354×10^{-7}
42	GO:BP	GO:0010212	response to ionizing radiation	3.106×10^{-7}
43	GO:BP	GO:0006997	nucleus organization	1.889×10^{-6}
44	GO:BP	GO:0051098	regulation of binding	1.935×10^{-6}
45	GO:BP	GO:0032728	positive regulation of interferon-beta production	3.461×10^{-6}
46	GO:BP	GO:0009411	response to UV	5.742×10^{-6}
47	GO:BP	GO:0006361	transcription initiation at RNA polymerase I promo...	8.197×10^{-6}
48	GO:BP	GO:0060338	regulation of type I interferon-mediated signaling ...	7.641×10^{-5}
49	GO:BP	GO:0034587	piRNA processing	2.768×10^{-4}
50	GO:BP	GO:0034475	U4 snRNA 3'-end processing	1.154×10^{-3}
51	GO:BP	GO:0070922	RISC complex assembly	1.573×10^{-3}
52	GO:BP	GO:0016925	protein sumoylation	2.296×10^{-3}
53	GO:BP	GO:0051145	smooth muscle cell differentiation	7.751×10^{-3}
54	GO:BP	GO:0040007	growth	1.199×10^{-2}
55	GO:BP	GO:0072079	nephron tubule formation	2.628×10^{-2}
56	GO:BP	GO:0001656	metanephros development	2.659×10^{-2}
57	GO:BP	GO:0048935	peripheral nervous system neuron development	4.027×10^{-2}
58	GO:BP	GO:0044355	clearance of foreign intracellular DNA	4.551×10^{-2}
59	GO:BP	GO:0000389	mRNA 3'-splice site recognition	4.551×10^{-2}
60	GO:BP	GO:0009648	photoperiodism	4.697×10^{-2}
61	GO:BP	GO:1903608	protein localization to cytoplasmic stress granule	4.990×10^{-2}
62	GO:CC	GO:0005654	nucleoplasm	4.941×10^{-324}
63	GO:CC	GO:0000785	chromatin	4.941×10^{-324}
64	GO:CC	GO:0035770	ribonucleoprotein granule	6.642×10^{-90}
65	GO:CC	GO:0044391	ribosomal subunit	3.175×10^{-64}
66	GO:CC	GO:0009295	nucleoid	2.951×10^{-12}
67	GO:CC	GO:0070993	cytosolic translation preinitiation complex	2.464×10^{-8}
68	GO:CC	GO:0034518	RNA cap binding complex	7.317×10^{-7}
69	GO:CC	GO:0120115	Lsm2-8 complex	8.264×10^{-4}
70	GO:CC	GO:1990726	Lsm1-7-Pat1 complex	3.467×10^{-2}
71	GO:CC	GO:0034719	SMN-Sm protein complex	3.992×10^{-2}

version e113_eg59_p19_6be52918
date 3/18/2026, 10:29:55 AM
organism hsapiens

g:Profiler

Figure S9: g:Profiler Manhattan plot for non-amyloidogenic human NABPs (n = 3816). Colour scheme and bubble encoding are identical to Figure S8. The enrichment landscape is dominated by broad nucleic-acid-metabolism terms and general transcription regulation, contrasting with the focused transcription-factor-activity signal seen in the amyloidogenic subset. Analysis run: g:Profiler version `e113_eg59_p19_6be52918`, 18 March 2026, organism: *Homo sapiens*.

Table S4: Top g:Profiler enrichment terms for non-amyloidogenic human NABPs (n = 3816). Note that many p_{adj} values are at machine-precision minimum ($\approx 4.94 \times 10^{-324}$), reflecting extremely large query size and near-complete term coverage. Columns as in Table S3.

Rank	Source	Term name	Term ID	p_{adj}
Molecular Function (GO:MF)				
1	GO:MF	transcription regulator activity	GO:0140110	$< 10^{-300}$
2	GO:MF	nucleic acid binding	GO:0003676	$< 10^{-300}$
3	GO:MF	DNA binding	GO:0003677	$< 10^{-300}$
4	GO:MF	double-stranded DNA binding	GO:0003690	$< 10^{-300}$
5	GO:MF	RNA binding	GO:0003723	$< 10^{-300}$
6	GO:MF	sequence-specific double-stranded DNA binding	GO:1990837	$< 10^{-300}$
7	GO:MF	DNA-binding transcription factor activity	GO:0003700	$< 10^{-300}$
8	GO:MF	DNA-binding TF activity, RNA Pol II-specific	GO:0000981	$< 10^{-300}$
9	GO:MF	transcription cis-regulatory region binding	GO:0000976	$< 10^{-300}$
10	GO:MF	RNA Pol II transcription regulatory region sequence-specific DNA binding	GO:0000977	$< 10^{-300}$
Biological Process (GO:BP)				
1	GO:BP	regulation of RNA metabolic process	GO:0051252	$< 10^{-300}$
2	GO:BP	macromolecule metabolic process	GO:0043170	$< 10^{-300}$
3	GO:BP	nucleobase-containing compound biosynthetic process	GO:0034654	$< 10^{-300}$
4	GO:BP	RNA biosynthetic process	GO:0032774	$< 10^{-300}$
5	GO:BP	regulation of metabolic process	GO:0019222	$< 10^{-300}$
6	GO:BP	regulation of nucleobase-containing compound metabolic process	GO:0019219	$< 10^{-300}$
7	GO:BP	RNA metabolic process	GO:0016070	$< 10^{-300}$
8	GO:BP	nucleobase-containing compound metabolic process	GO:0006139	$< 10^{-300}$
9	GO:BP	DNA-templated transcription	GO:0006351	$< 10^{-300}$
10	GO:BP	regulation of DNA-templated transcription	GO:0006355	$< 10^{-300}$

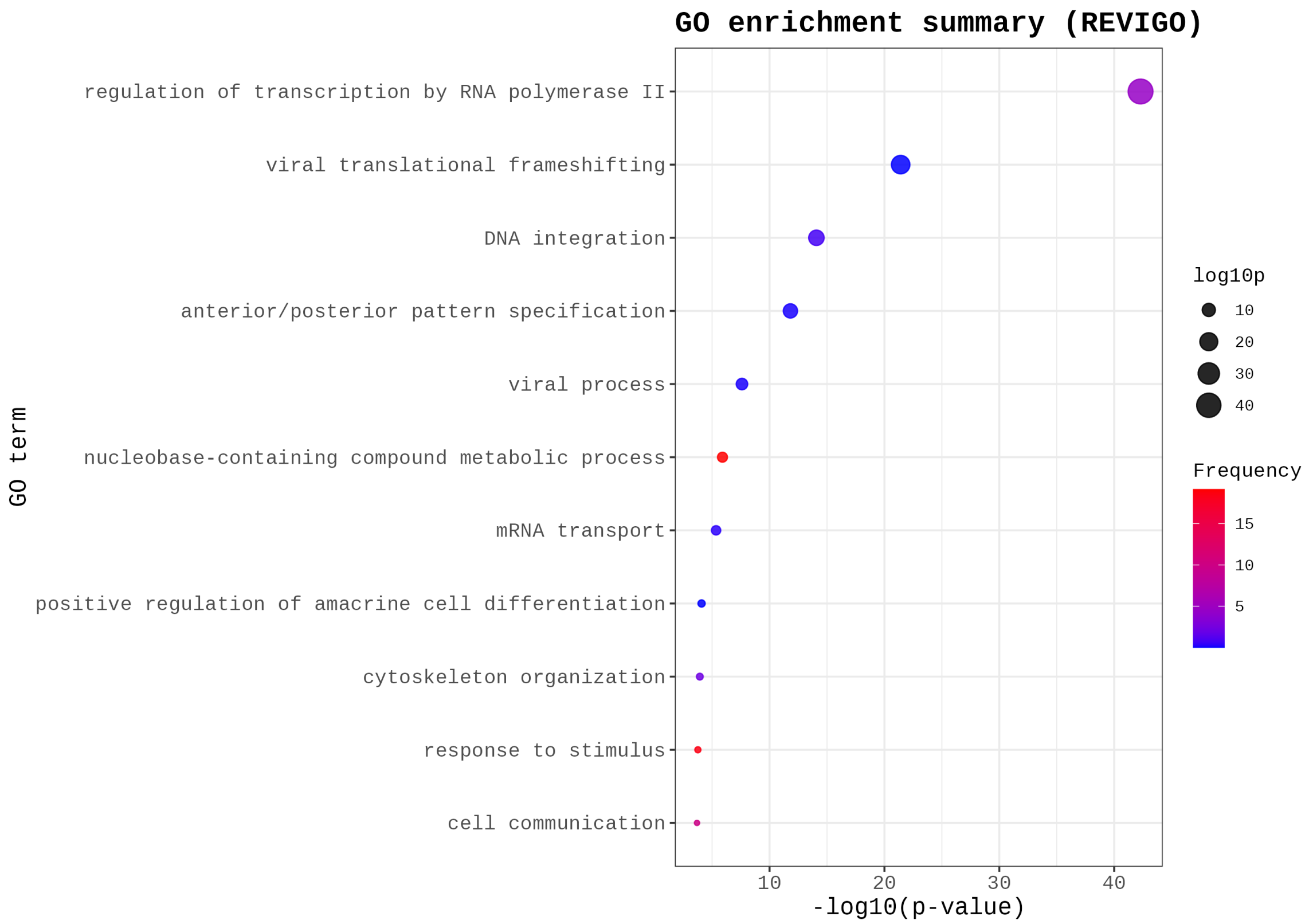

Figure S10: REVIGO semantic clustering of enriched GO biological process terms for amyloidogenic human NABPs (n = 189). Each bubble represents a semantically non-redundant representative GO term after REVIGO collapsed the full g:Profiler result set using SimRel similarity scores. Bubble size encodes $-\log_{10}(p\text{-value})$ (larger bubbles = more significant); bubble colour encodes term frequency in the Gene Ontology graph (warm/red = high-frequency broad parent terms; cool/blue−purple = low-frequency specific child terms). The dominant term — *regulation of transcription by RNA polymerase II* — is both the most significant ($-\log_{10} p \approx 43$) and a relatively specific term (blue/purple), confirming that the transcriptional regulatory signal is not an artefact of broad, uninformative parent terms. Crucially, this signal survives REVIGO's aggressive redundancy removal, which constitutes the strongest possible evidence that transcriptional regulation is the genuine, non-redundant functional hallmark of amyloidogenic NABPs.

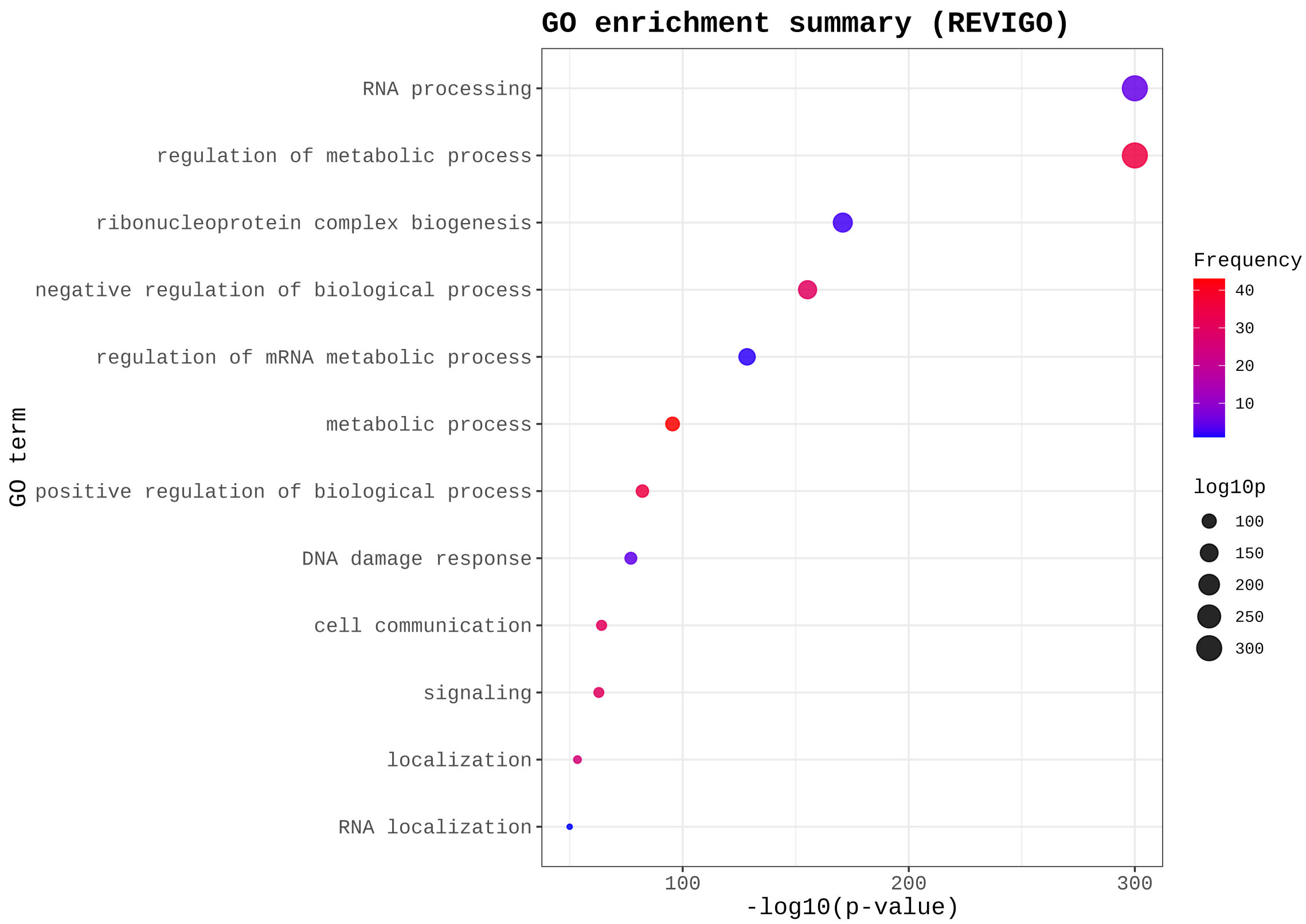

Figure S11: REVIGO semantic clustering of enriched GO biological process terms for non-amyloidogenic human NABPs (n = 3816). Encoding as in Figure S10. Note the substantially larger $-\log_{10} p$ axis scale (extending to ∼300 versus ∼43 for the amyloidogenic set), reflecting the much larger query size. After redundancy removal, the non-amyloidogenic set is characterised by broad metabolic and RNA-processing themes — *RNA processing* and *regulation of metabolic process* dominate — which represent expected background functions of the full nucleic-acid-binding proteome. The focused transcription-factor regulatory signature that defines the amyloidogenic subset is conspicuously absent here as a dominant isolated cluster, underscoring its specificity to the amyloidogenic fraction. The warm colouration (red/pink) of the two top terms indicates these are high-frequency, broad ontological categories, whereas the leading amyloidogenic term is a more specific cool-coloured node, further reinforcing the functional specificity of the amyloidogenic enrichment signal.

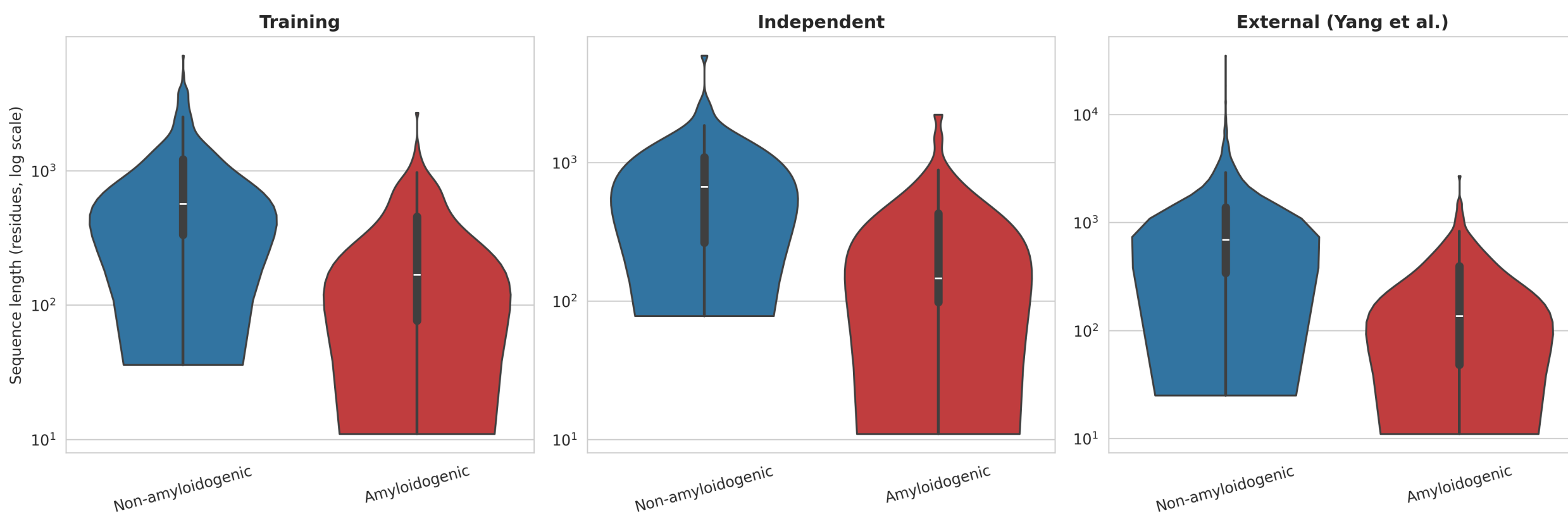

Figure S12: Sequence length distributions differ substantially between amyloidogenic and non-amyloidogenic classes across all evaluated datasets. Violin plots (log-scale y-axis) show sequence length distributions for amyloidogenic and non-amyloidogenic proteins in the training set (including extremophile-derived negatives), independent test set, and external test set (Yang et al.). Median lengths, effect sizes, and statistical significance for each comparison are reported in Supplementary Table S10.

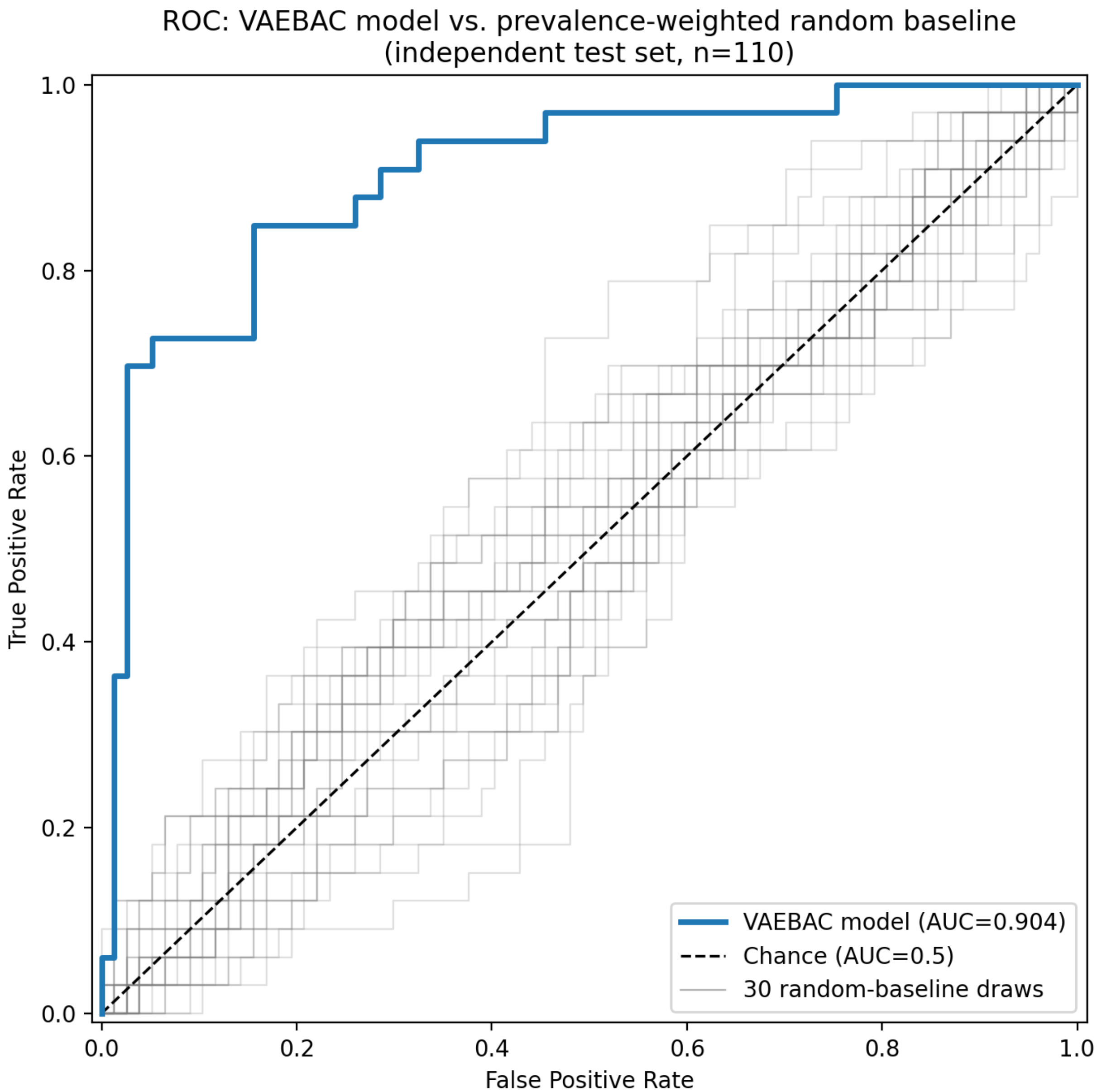

Figure S13: VAEBAC substantially exceeds properly-defined random classifier baselines. ROC curve for VAEBAC (blue; AUC = 0.904) shown alongside 30 individual ROC curves from a prevalence-weighted random classifier (gray, random uniform scores) and the theoretical random reference (dashed; AUC = 0.5). A majority-class baseline (always predicting the non-amyloidogenic class) achieves 70.0% accuracy with MCC = 0, illustrating that accuracy alone is a misleading metric under class imbalance; VAEBAC's MCC (0.660) lies well outside the full spread of the prevalence-weighted random baseline (mean = 0.000, SD = 0.096). Full numerical comparison is provided in Supplementary Table S11.

Supplementary Tables — Performance and CD Data

Table S5: Secondary structure content estimated by BeStSel at multiple time points during *in vitro* aggregation assays.

Protein	Time (h)	α-Helix (%)	Antipar. β (%)	Par. β (%)	Turn (%)	Others (%)
P0AA04	0	8.3	20.6	8.3	14.6	48.2
P0AA04	12	0.0	34.6	0.0	13.4	52.0
P0AA04	24	0.0	43.0	0.0	12.9	44.1
P0AA04	48	0.0	38.4	0.0	14.0	47.6
P0AA04	72	0.0	40.2	0.0	14.1	45.7
P0AA04	96	0.0	43.0	0.0	20.5	36.5
P0C037	0	0.0	33.8	0.0	21.4	44.8
P0C037	6	0.0	37.2	7.6	12.4	42.8
P0C037	12	0.0	40.9	0.0	15.7	43.4
P0C037	18	0.0	41.5	0.0	13.7	44.8
B1XF10	0	0.0	40.6	0.0	14.3	45.1
B1XF10	3	0.0	41.2	0.0	14.9	44.0
B1XF10	6	0.3	37.8	0.0	15.0	46.9
B1XF10	9	0.0	39.4	0.0	14.4	46.2

Table S6: Comparative performance of VAEBAC and FA-AMY under standard and homology-controlled external validation settings. Performance metrics include Matthews correlation coefficient (MCC), area under the ROC curve (AUC), balanced accuracy (BA), and F1-score. Retention (%) represents the ratio of external MCC to internal cross-validation MCC, indicating model stability under independent evaluation. Homology-controlled evaluation was performed following sequence clustering at 40% identity using CD-HIT to prevent homologous overlap between training and test sets. Bold values indicate the best result for each metric where a direct comparison is available. [a]Evaluated on a homology-clustered dataset (CD-HIT, 40% sequence identity threshold). [b]FA-Amy's internal MCC shown here is its general (non-homology-specific) cross-validation performance, reused from the standard comparison above for reference; a homology-split-specific internal MCC was not separately computed for either model. [c]Not reported: under the homology-controlled split, only VAEBAC's external MCC was directly evaluated; balanced accuracy, F1-score, and AUC were not computed for VAEBAC in this setting, and a matched internal MCC is not available for either model under this specific split, so no retention percentage is reported for this comparison (see main text). [d]Input handling: for comparability with the FA-Amy evaluation protocol, external sequences were truncated to their first 1,000 residues prior to evaluation and any residue outside the model's 23-character alphabet was recoded as `X`. All 1,107 Yang et al. sequences were retained; truncation affected 338 sequences (30.5%; 7 positive, 331 negative) and recoding affected 1 sequence. This differs from the primary external evaluation reported in the main text, in which 6 over-length and 1 selenocysteine-containing negative were excluded ($n = 1{,}100$); the two analyses are reported separately and are not directly comparable. Abbreviations: BA, balanced accuracy; Ext., external dataset.

Model	Internal MCC	External MCC	Retention (%)	AUC (Ext.)	BA (Ext.)	F1 (Ext.)
FA-Amy	0.842	0.492	58.4%	0.918	0.832	0.540
VAEBAC	0.730	**0.657**	**90.0%**	0.914	**0.851**	**0.709**
FA-AMY[a]	0.842[b]	0.606	–[c]	0.939	0.879	0.648
VAEBAC[a]	–[c]	**0.744**	–[c]	–[c]	–[c]	–[c]

Table S7: Cross-validation performance across CD-HIT sequence identity thresholds. The classifier head was retrained from initialization (with the pretrained VAE encoder frozen) at each threshold and evaluated via 5-fold stratified cross-validation. To ensure a consistent frozen encoder across all threshold comparisons, this analysis used an independently-trained checkpoint sharing identical encoder architecture and weights to the primary reported model (Methods); reported values are therefore not directly numerically comparable to Table 2, but the relative trend across thresholds is the relevant comparison for assessing redundancy sensitivity. Values represent mean ± SD across folds.

CD-HIT Threshold	Sequences Retained	Accuracy (mean ± SD)	MCC (mean ± SD)	AUC (mean ± SD)
Unfiltered (baseline)	408/408	0.880 ± 0.040	0.721 ± 0.099	0.942 ± 0.027
90% identity	395/408	0.868 ± 0.033	0.700 ± 0.079	0.936 ± 0.023
70% identity	374/408	0.885 ± 0.028	0.739 ± 0.063	0.941 ± 0.018
50% identity	361/408	0.887 ± 0.010	0.731 ± 0.022	0.941 ± 0.015
40% identity	348/408	0.879 ± 0.041	0.721 ± 0.098	0.941 ± 0.023

Table S8: Comparison against a position-specific scoring matrix (PSSM)-based representation. PSSMs were generated via PSI-BLAST (3 iterations, E-value < 0.001) against the Swiss-Prot database for all training and independent test sequences; 56 of 518 sequences (47 negative, 9 positive) yielded no detectable homologs at this threshold and were excluded. Amino-acid-composition PSSM (AAC-PSSM) features (20-dimensional, column-averaged log-odds scores) were used to train a classifier of comparable capacity to the primary VAEBAC classifier head under an identical cross-validation protocol. Independent test set metrics for VAEBAC are reported on the complete 110-sequence benchmark at the Youden J-optimal threshold ($\tau = 0.43919$; matching Table 3); AAC-PSSM metrics are reported on the 93 of 110 sequences for which a PSSM could be generated (17 excluded: 4 positive, 13 negative).

**Representation**	**Accuracy**	**MCC**	**AUC**
5-fold cross-validation (training set)			
One-hot + physicochemical (VAEBAC)	0.875 ± 0.021	0.713 ± 0.054	0.944 ± 0.019
AAC-PSSM	0.791 ± 0.028	0.558 ± 0.049	0.829 ± 0.033
Independent test set			
One-hot + physicochemical (VAEBAC)	0.846	0.660	0.904
AAC-PSSM	0.774	0.527	0.830

Table S9: Latent representation dimensionality and VAE bottleneck ablation. A lightweight probe classifier (comparable capacity to the primary VAEBAC classifier head, with dropout and L2 regularization) was trained via 5-fold cross-validation on the pretrained VAE encoder's 512-dimensional latent representation (μ), on PCA-reduced projections of that representation, and on pre-bottleneck pooled features (bypassing the VAE encoder entirely). PCA component 1 (94.8% of latent variance) was found to correlate strongly with sequence length (Pearson $r = 0.990$; see Discussion); results are reported both with and without this component to isolate genuine amyloid-relevant signal from a length-driven representational artifact.

Representation	Accuracy	MCC	AUC
Full latent space (512-dim, includes length-correlated PC1)			
512 (full μ)	0.789 ± 0.031	0.575 ± 0.049	0.894 ± 0.025
256 (PCA)	0.606 ± 0.078	0.047 ± 0.141	0.524 ± 0.063
128 (PCA)	0.667 ± 0.042	0.216 ± 0.092	0.675 ± 0.056
64 (PCA)	0.728 ± 0.037	0.368 ± 0.091	0.774 ± 0.032
32 (PCA)	0.792 ± 0.037	0.524 ± 0.086	0.862 ± 0.025
PCA excluding length-correlated PC1 (406-dim source)			
406 (no PC1)	0.561 ± 0.028	-0.240 ± 0.035	0.294 ± 0.034
256 (no PC1)	0.522 ± 0.039	-0.125 ± 0.057	0.428 ± 0.053
128 (no PC1)	0.584 ± 0.064	0.019 ± 0.121	0.524 ± 0.057
64 (no PC1)	0.669 ± 0.020	0.206 ± 0.051	0.674 ± 0.056
32 (no PC1)	0.713 ± 0.030	0.344 ± 0.055	0.760 ± 0.054
Pre-bottleneck features (bypasses VAE entirely)			
24 (pre-VAE pooled)	0.750 ± 0.023	0.436 ± 0.066	0.808 ± 0.029

Table S10: Sequence length distribution between amyloidogenic (positive) and non-amyloidogenic (negative) classes across training, independent, and external test datasets. Statistical comparisons use the Mann-Whitney U test; effect size is reported as Cohen's d. Length-only AUC reflects logistic regression using sequence length as the sole predictor, evaluated via repeated (20×) 5-fold stratified cross-validation. Length-only AUC was not computed for the training set, as class-wise separability is already directly quantified via the Mann-Whitney test and Cohen's d for this dataset; length-only AUC is instead reported for the independent and external test sets to assess generalization of this signal to held-out data.

Dataset	n	Pos. median	Neg. median	Mann-Whitney p	Cohen's d	Length-only AUC
Training set (incl. extremophile negatives)	408	168	564	2.94×10^{-19}	-0.720	–
Independent test set	110	146	692	2.22×10^{-6}	-0.564	0.789 ± 0.110
External test set (Yang et al.)	1100	136	687	2.03×10^{-40}	-0.690	0.828 ± 0.036

Table S11: Comparison of VAEBAC against random-classifier and label-permutation baselines on the independent test set ($n = 110$). The prevalence-weighted random baseline reflects 10,000 trials of random binary predictions sampled at the true class prevalence, with random continuous scores used for AUC estimation (mean ± SD reported). The label-permutation baseline reflects VAEBAC's own training procedure (classifier head retrained from initialization, encoder frozen) applied to randomly shuffled labels, evaluated by 5-fold cross-validation (mean ± SD reported).

**Method**	**Accuracy**	**MCC**	**AUC**
Majority-class baseline	0.700	0.000 (undefined)	–
Prevalence-weighted random (10,000 trials)	0.580 ± 0.044	0.000 ± 0.096	0.501 ± 0.060
Label-permutation baseline (5-fold CV)	0.582 ± 0.127	0.039 ± 0.283	0.537 ± 0.169
VAEBAC (actual model)	0.845	0.660	0.904

Table S12: Length-only baseline versus VAEBAC, and SHAP–length correlation of the classifier head, on the 110-sequence independent benchmark. **(A)** A logistic regression using true (unpadded) sequence length as its only predictor was fitted on the 318-sequence training set and applied unchanged to the independent benchmark. **(B)** The 20 latent dimensions ranked highest by mean |SHAP value| for the classifier head, with the Pearson correlation of each dimension against true sequence length; p-values are Bonferroni-corrected across all 512 latent dimensions. Across the full latent space the median is $|r| = 0.96$ and 95.1% of dimensions exceed $|r| = 0.3$ (Supplementary Figure S16).

(A) Discriminative performance

**Model**	**Threshold**	**Accuracy**	**Sensitivity**	**Specificity**	**MCC**	**AUC**
VAEBAC	calibrated ($\tau = 0.9354$)	0.891	0.697	0.974	0.734	0.904
VAEBAC	Youden ($\tau = 0.4392$)	0.845	0.848	0.844	0.660	0.904
Length-only LR	Youden	0.700	0.848	0.636	0.444	0.786
Length-only LR	0.5	0.673	0.697	0.662	0.331	0.786

DeLong's test, VAEBAC versus length-only: $z = 2.775$, $p = 0.0055$. Likelihood-ratio test of label $\sim$ length + VAEBAC score against label $\sim$ length: LR = 37.32, df = 1, $p = 1.0 \times 10^{-9}$ (VAEBAC coefficient = 4.44; length coefficient = −0.34). Length-only 5-fold cross-validated AUC on the training set: 0.810 ± 0.054.

(B) Top-20 SHAP dimensions versus sequence length

**Rank**	**Latent dim.**	**Mean |SHAP|**	**Pearson r**	**p (Bonferroni)**
1	491	0.0855	0.904	7.6×10^{-39}
2	352	0.0705	0.667	9.1×10^{-13}
3	151	0.0663	0.938	1.1×10^{-48}
4	390	0.0640	−0.917	2.6×10^{-42}
5	426	0.0576	0.822	1.9×10^{-25}
6	387	0.0558	0.404	6.2×10^{-3}
7	364	0.0530	−0.776	1.2×10^{-20}
8	272	0.0517	0.962	1.1×10^{-59}
9	136	0.0516	−0.930	4.6×10^{-46}
10	321	0.0510	−0.490	2.9×10^{-5}
11	17	0.0490	0.987	1.2×10^{-85}
12	461	0.0486	−0.914	1.7×10^{-41}
13	145	0.0482	−0.736	2.7×10^{-17}
14	495	0.0475	−0.968	4.0×10^{-64}
15	163	0.0462	0.823	1.3×10^{-25}
16	186	0.0452	−0.253	1.000
17	325	0.0445	−0.912	5.6×10^{-41}
18	230	0.0427	−0.955	6.3×10^{-56}
19	293	0.0423	−0.513	5.4×10^{-6}
20	106	0.0408	0.946	9.4×10^{-52}

Table S13: Length-stratified performance of VAEBAC on the 110-sequence independent benchmark. Sequences were partitioned into quartiles by true (unpadded) length and evaluated at the fixed benchmark-calibrated threshold ($\tau = 0.9354$). MCC confidence intervals are bootstrap percentile intervals (1,000 resamples). Quartiles Q3 and Q4 contain fewer than five positive sequences, so MCC is unstable and is not reported for them; AUC is given instead.

Quartile	Length (res.)	n	Pos.	Neg.	Accuracy	MCC (95% CI)	AUC
Q1	11–157	28	18	10	0.893	0.801 (0.596–1.000)	0.917
Q2	168–451	27	9	18	0.815	0.574 (0.189–0.892)	0.809
Q3	459–972	27	4	23	0.926	not reported ($<$ 5 pos.)	0.902
Q4	1,070–10,746	28	2	26	0.929	not reported ($<$ 5 pos.)	0.731

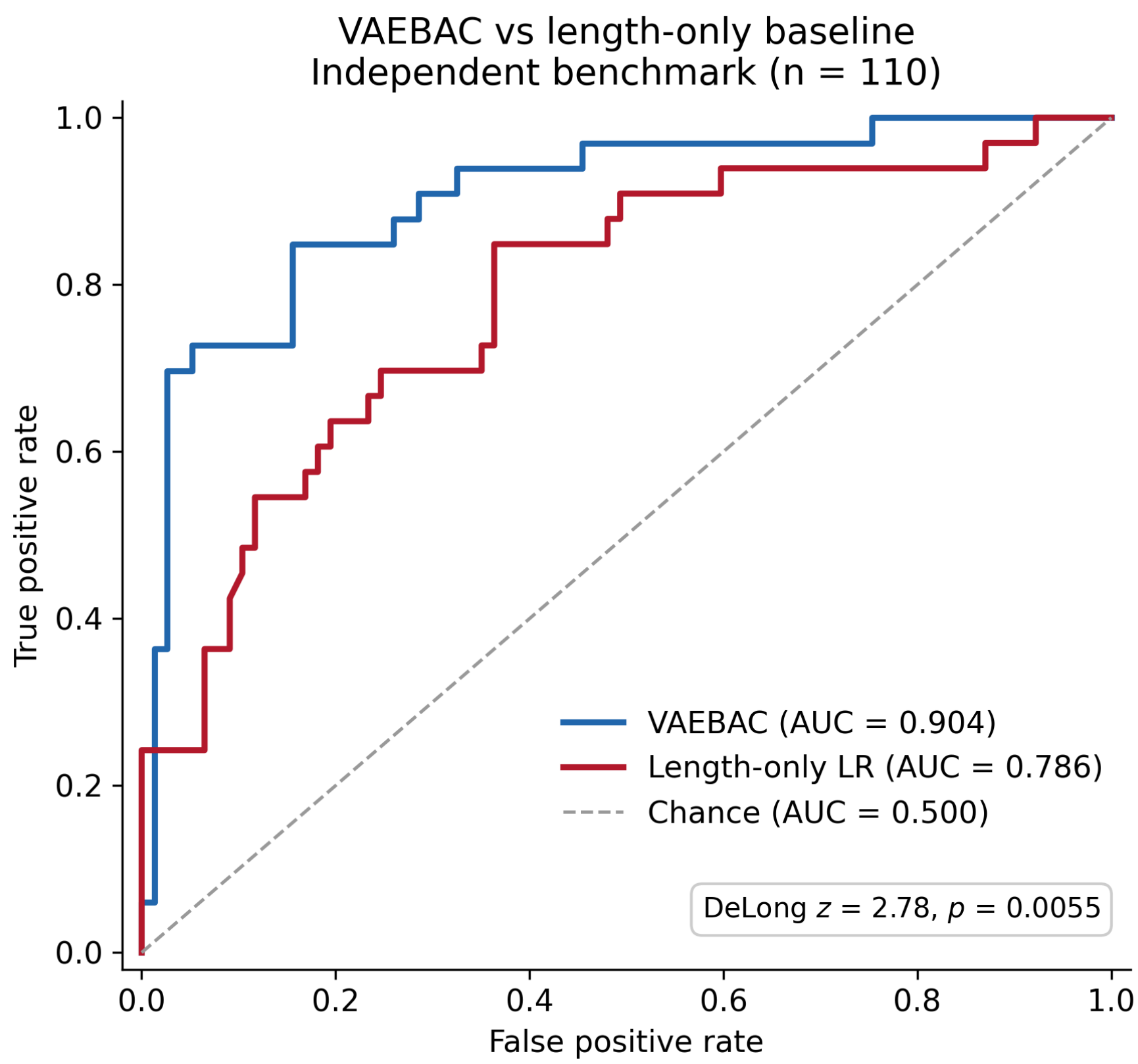

Figure S14: VAEBAC versus a length-only baseline on the 110-sequence independent benchmark. ROC curves for VAEBAC (AUC = 0.904) and for a logistic regression using true sequence length as its sole predictor (AUC = 0.786), fitted on the 318-sequence training set and applied unchanged. The AUC difference is significant by DeLong's test ($z = 2.775$, $p = 0.0055$). Corresponding metrics are given in Supplementary Table S12A.

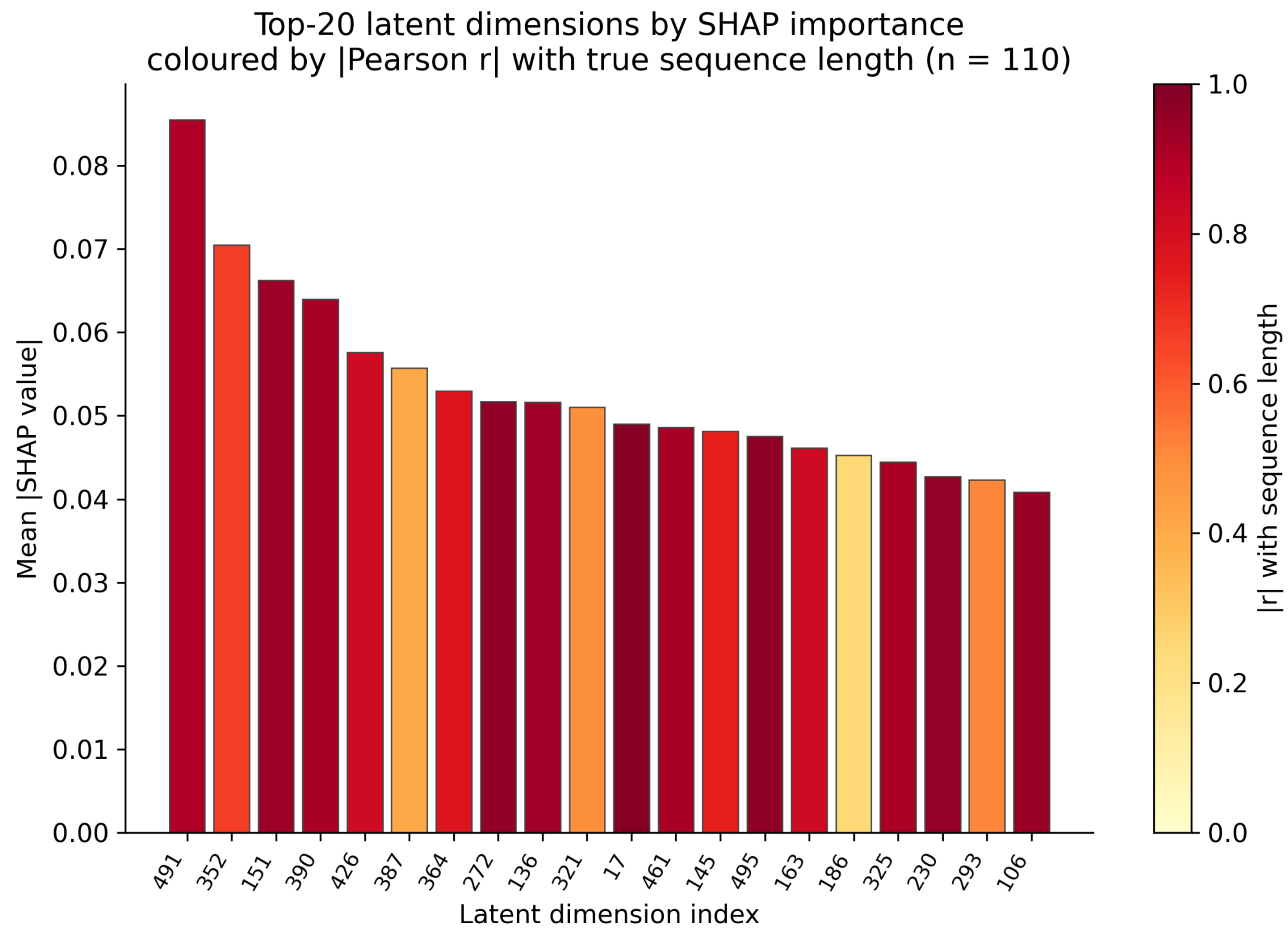

Figure S15: The latent dimensions most influential to the classifier head are length-correlated. The 20 latent dimensions with the highest mean |SHAP value| on the independent benchmark, coloured by the absolute Pearson correlation of each dimension with true sequence length. Nineteen of the 20 exceed $|r| = 0.3$ (median $|r| = 0.91$). Values are listed in Supplementary Table S12B.

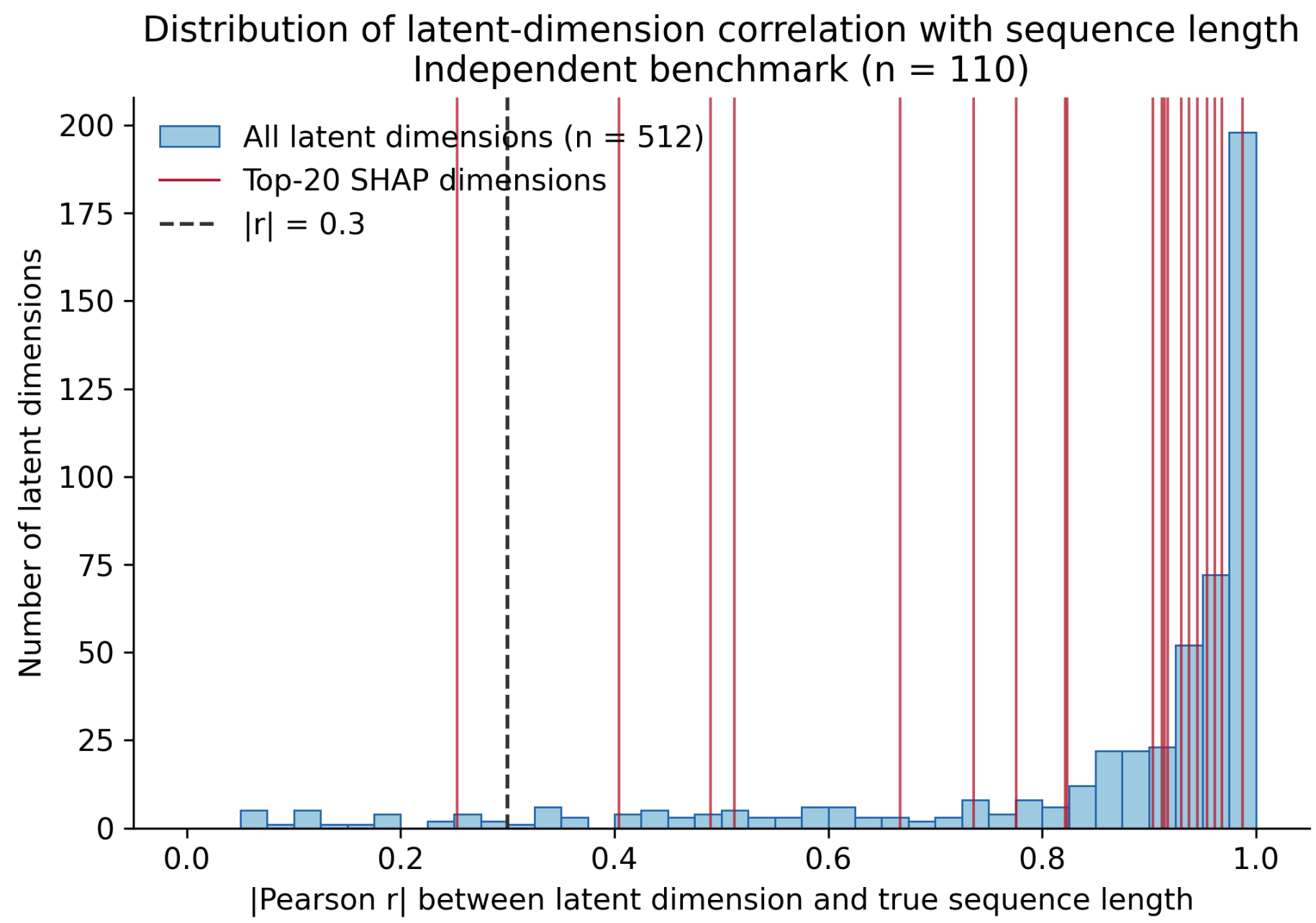

Figure S16: Distribution of latent-dimension correlation with sequence length across the full 512-dimensional latent space. Histogram of |Pearson r| between each latent dimension and true sequence length on the independent benchmark; red lines mark the 20 highest-SHAP dimensions and the dashed line marks $|r| = 0.3$. The median across all dimensions is $|r| = 0.96$, and 95.1% exceed $|r| = 0.3$.

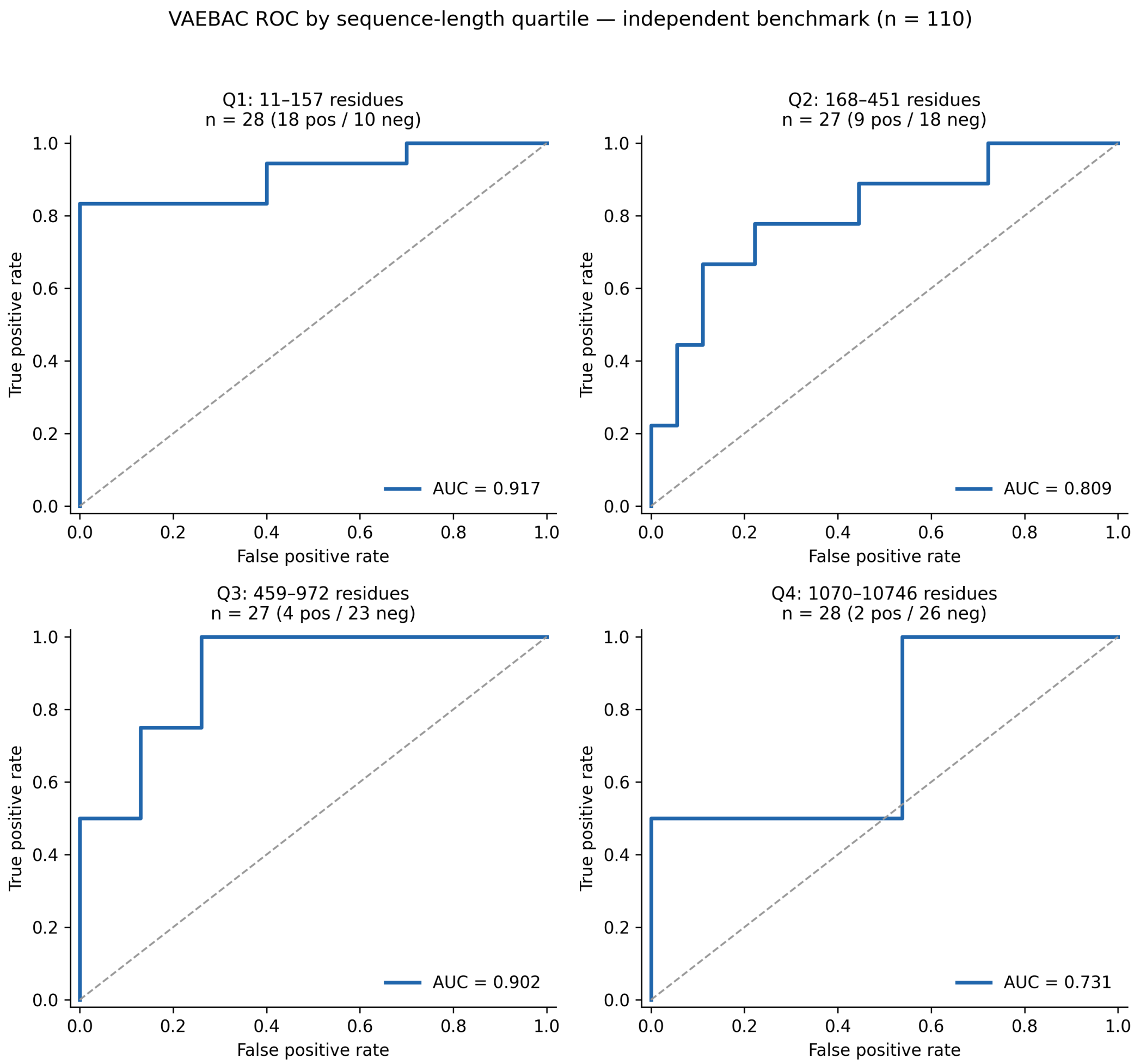

Figure S17: VAEBAC ROC curves by sequence-length quartile, independent benchmark. One panel per quartile of true sequence length. Discrimination remains above chance in every stratum (AUC 0.731–0.917) but is weakest in the longest quartile. Panel sample sizes and class balance are given in Supplementary Table S13.

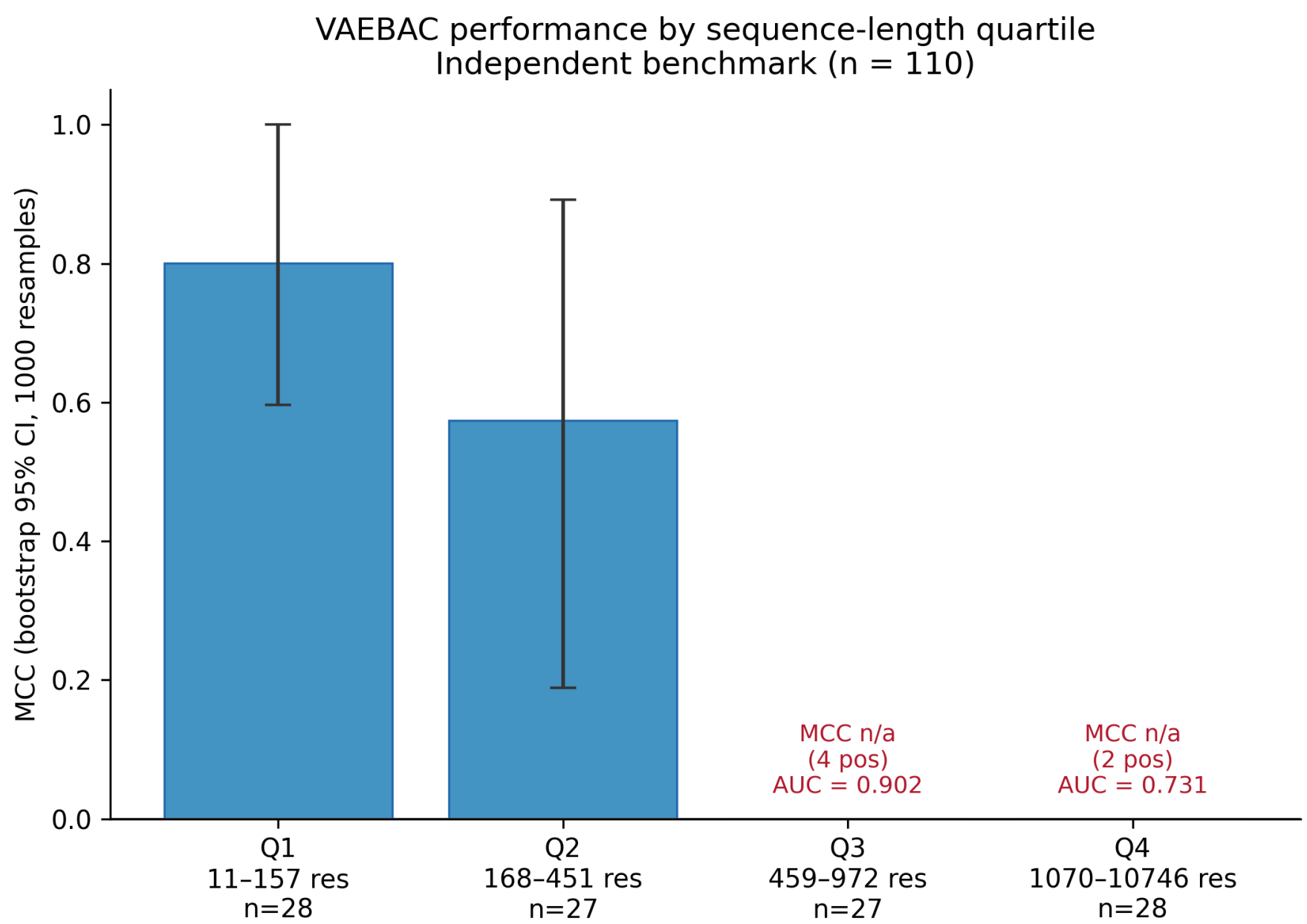

Figure S18: MCC by sequence-length quartile with bootstrap 95% confidence intervals, independent benchmark. Intervals are percentile intervals from 1,000 bootstrap resamples. MCC is omitted for Q3 and Q4, which contain only four and two positive sequences respectively; AUC is annotated instead.

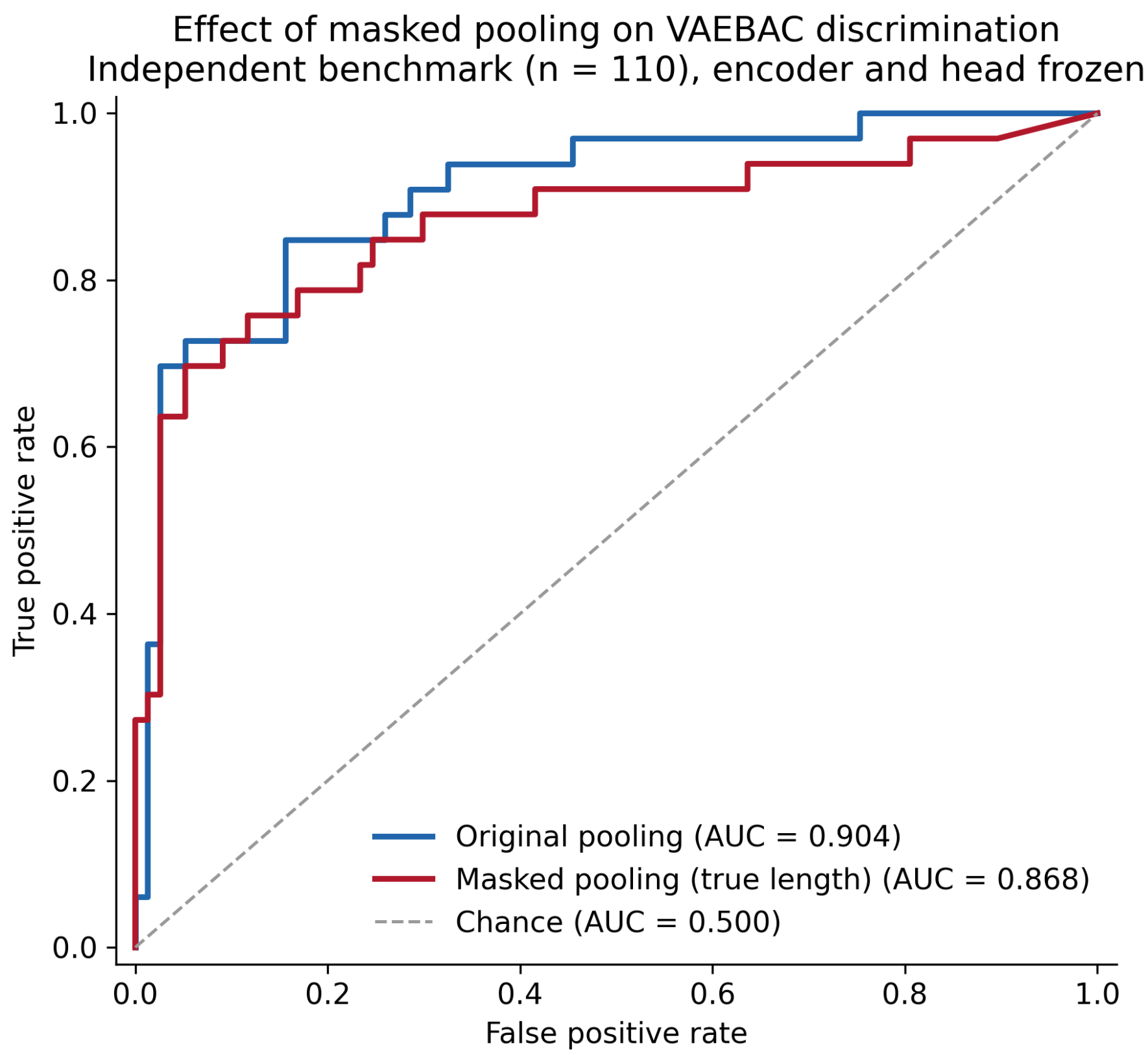

Figure S19: Masked mean pooling removes the length channel at a small cost in ranking performance. ROC curves on the 110-sequence independent benchmark for the shipped encoder (adaptive average pooling across the full padded width of 10,746 positions; AUC = 0.904) and for the same frozen encoder and classifier head with pooling restricted to each sequence's true unpadded length (AUC = 0.868). No weights were retrained. Under masked pooling the correlation between the dominant latent principal component and sequence length falls from $|r| = 0.990$ to $|r| = 0.025$, the median absolute correlation of the 20 highest-SHAP latent dimensions with length falls from 0.908 to 0.028, and no such dimension remains significantly correlated with length after Bonferroni correction. Matthews correlation coefficient at each curve's Youden-optimal operating point improves from 0.660 to 0.687. Note that masked pooling shifts the latent scale the classifier head was fitted under, so the resulting probabilities are not calibrated (all fall below 0.5) and only the ranking and the Youden-optimal operating point are interpretable.
